# Studying the Parkinson’s disease metabolome and exposome in biological samples through different analytical and cheminformatics approaches: a pilot study

**DOI:** 10.1007/s00216-022-04207-z

**Published:** 2022-07-13

**Authors:** Begoña Talavera Andújar, Dagny Aurich, Velma T. E. Aho, Randolph R. Singh, Tiejun Cheng, Leonid Zaslavsky, Evan E. Bolton, Brit Mollenhauer, Paul Wilmes, Emma L. Schymanski

**Affiliations:** 1 Luxembourg Centre for Systems Biomedicine (LCSB), University of Luxembourg, Avenue du Swing 6, 4367 Belvaux, Luxembourg; 2 IFREMER (Institut Français de Recherche Pour L’Exploitation de La Mer), Unité Contamination Chimique Des Ecosystèmes Marins, Nantes, France; 3 National Center for Biotechnology Information, National Library of Medicine, National Institutes of Health, Bethesda, MD 20894 USA; 4 Department of Neurology, University Medical Center Göttingen, Göttingen, Germany; 5 Paracelsus-Elena-Klinik, Kassel, Germany; 6 Department of Life Sciences and Medicine, Faculty of Science, Technology and Medicine, University of Luxembourg, Esch-sur-Alzette, Luxembourg

**Keywords:** Liquid chromatography (LC), Non-target high-resolution mass spectrometry (NT-HRMS), Metabolomics, Exposomics, Parkinson’s disease, Gut dysbiosis

## Abstract

Parkinson’s disease (PD) is the second most prevalent neurodegenerative disease, with an increasing incidence in recent years due to the aging population. Genetic mutations alone only explain <10% of PD cases, while environmental factors, including small molecules, may play a significant role in PD. In the present work, 22 plasma (11 PD, 11 control) and 19 feces samples (10 PD, 9 control) were analyzed by non-target high-resolution mass spectrometry (NT-HRMS) coupled to two liquid chromatography (LC) methods (reversed-phase (RP) and hydrophilic interaction liquid chromatography (HILIC)). A cheminformatics workflow was optimized using open software (MS-DIAL and patRoon) and open databases (all public MSP-formatted spectral libraries for MS-DIAL, PubChemLite for Exposomics, and the LITMINEDNEURO list for patRoon). Furthermore, five disease-specific databases and three suspect lists (on PD and related disorders) were developed, using PubChem functionality to identifying relevant unknown chemicals. The results showed that non-target screening with the larger databases generally provided better results compared with smaller suspect lists. However, two suspect screening approaches with patRoon were also good options to study specific chemicals in PD. The combination of chromatographic methods (RP and HILIC) as well as two ionization modes (positive and negative) enhanced the coverage of chemicals in the biological samples. While most metabolomics studies in PD have focused on blood and cerebrospinal fluid, we found a higher number of relevant features in feces, such as alanine betaine or nicotinamide, which can be directly metabolized by gut microbiota. This highlights the potential role of gut dysbiosis in PD development.

**Supplementary Information:**

The online version contains supplementary material available at 10.1007/s00216-022-04207-z.

## Introduction

Neurodegenerative diseases are characterized by the progressive degeneration of neuronal cells in the brain, with aging being the main risk factor for their development. However, most cases are sporadic and multifactorial in origin [1]. Parkinson’s disease (PD) is the second most prevalent neurodegenerative disease, with an increasing incidence in recent years due to the increasingly aging population. To date, PD is an incurable and progressive disorder characterized by the degeneration and loss of dopaminergic neurons in the *substantia nigra pars compacta* and the accumulation of intracytoplasmic inclusions, known as *Lewy bodies* [2–4]. Tremor, rigidity, bradykinesia, and postural instability are the most characteristic features of PD. Nevertheless, gastrointestinal (GI) symptoms are the predominant non-motor features in PD, including constipation (most common), dyspepsia, gastroparesis, bloating, or dysphagia. Other non-motor symptoms found in PD patients are hyposmia and REM-sleep behavior disorder (RBD), as well as cognitive, neuropsychiatric, autonomic, and sensory disturbances [5, 6].

The current hypotheses for the pathogenesis of PD include protein misfolding and aggregation, mitochondrial injury, oxidative stress, and inflammation [2]. The abnormal accumulation of misfolded alpha-synuclein (α-Syn) protein inside the neurons leads to the formation of Lewy bodies. Normally, aggregations of α-Syn should be cleared by adequately functioning Leucine-Rich Repeat Kinase 2 (LRRK2) activity, which delays the progression of PD. Thus, genetic mutations of LRRK2 are known as a risk factor of PD. Nevertheless, since genetic mutations alone only clarify less than 10% of PD cases, environmental factors such as exposure to metals, pesticides, and drugs may play a role in up to 90% of PD cases [4]. At least 5 studies have reported a marked PD risk after exposure to paraquat, which is one of the most widely used pesticides in the world [7].

**Fig. 1:**
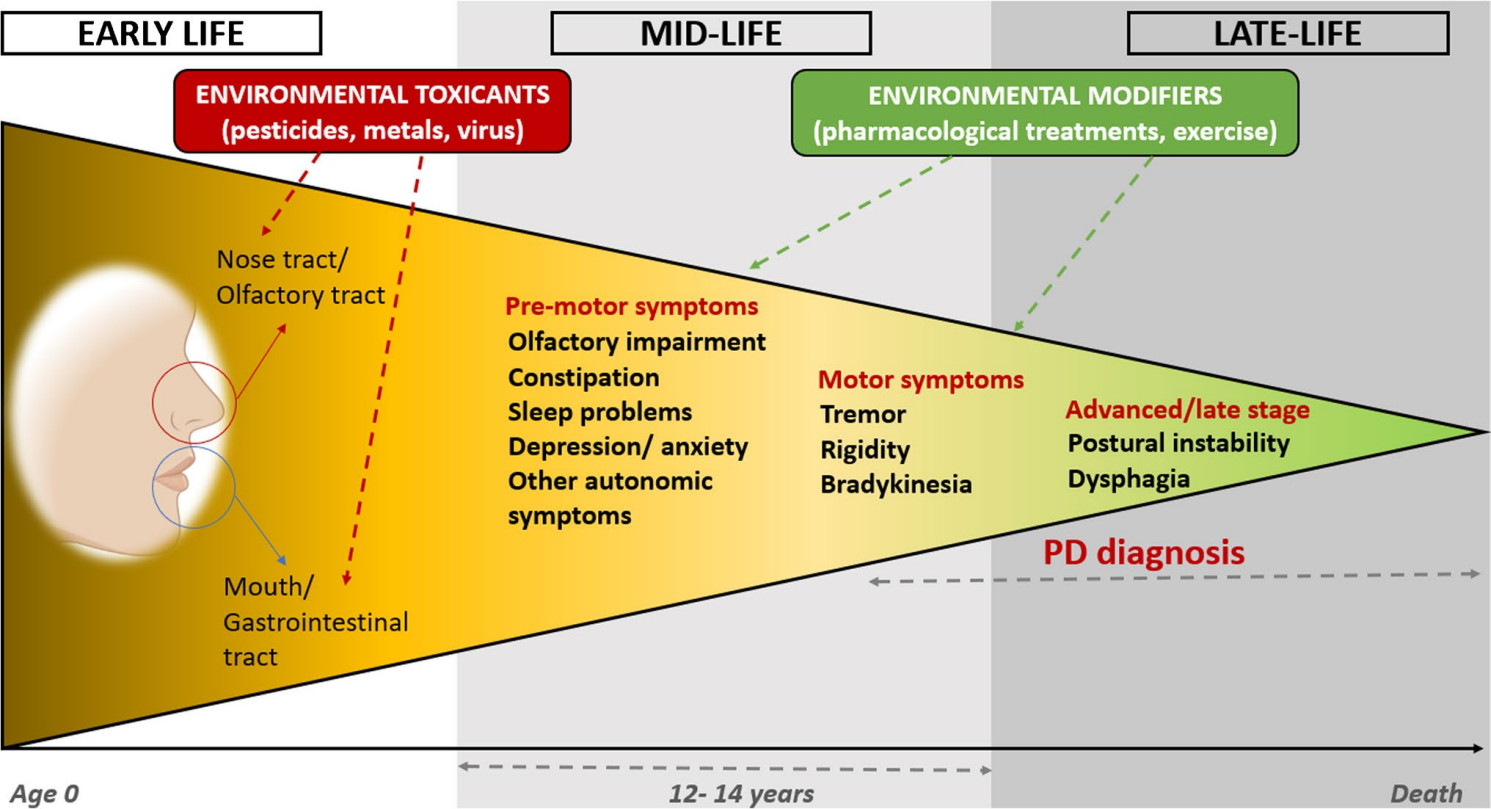
Environmental toxicants, viruses, or other agents could enter the human body (via mouth or nose) inducing pathological α-Syn aggregations in susceptible individuals via inflammation or microbiome dysbiosis. Over years, the pathology may progress to the central olfactory structures and/or lower brain, leading to a pre-motor or prodromal period (early-midlife) characterized by rather unspecific symptoms (e.g., constipation or sleep disorders). This period could have a duration of 12–14 years. Next, some individuals may develop motor symptoms (e.g., tremor or rigidity) that could lead to PD diagnosis. Finally, in the advanced/late period, axial motor symptoms like postural instability tend to occur [8, 9, 14]. Picture adapted from Honglei Chen et al. [9]

According to the Braak hypothesis [8] (Fig. 1), environmental factors could induce pathological α-Syn accumulation via the olfactory or gastrointestinal tract, where over time the pathology may progress to the central nervous system (CNS), resulting in symptoms such as sleep disturbance or motor deficits. This might be explained by the bidirectional interaction between the gut microbiome and the CNS, known as the “microbiota-gut-brain axis” (MGBA). Therefore, the dysregulation of the MGBA could lead to the spread of α-Syn from the gut to the brain through the vagal nerve, which could explain PD neurodegeneration [1, 5, 9]. Previous studies reported differentially abundant gut microbes in PD patients compared to the healthy control (Ctrl) group, such as increases in the genus *Akkermansia* [10], or reduction in the *Lachnospiraceae* family, which is associated with anti-inflammatory and neuroprotective effects [11, 12]. Alterations in gut microbiota composition and function, known as gut dysbiosis, may increase intestinal and blood–brain barrier permeabilities, allowing the accumulation of pro-inflammatory molecules in the brain. As gut dysbiosis occurs in the early stages of the disease, the characterization and, where necessary, prompt modification of the gut microbiota (e.g., via pro- or prebiotic treatments) may be a promising therapeutic approach [5, 13].

Currently, the diagnosis of PD mainly relies on clinical symptoms, medical history, and response to dopaminergic treatment, which results in a high rate of misdiagnosis due to the lack of motor symptoms during the early disease stages and the rather unspecific premotor symptoms [2, 3, 15]. Nowadays, dopamine replacement therapy is the “gold standard” treatment in PD. However, these types of drugs can only improve the motor symptoms and are incapable of slowing or halting neurodegeneration and thus disease progression. Therefore, research is urgently needed to identify environmental contributions to PD development as well as endogenous biomarkers, both of which could help the earlier recognition, prevention, and/or treatment of the disease [2, 3, 16].

One approach to accomplish this challenge is through the study of the exposome and metabolome in biological samples of PD patients, such as blood, feces, or urine. The *exposome* is defined as a measure of the totality of human environmental exposures over an individual’s lifetime; meanwhile, the *metabolome* represents the collection of small compound metabolites in the organism, typically under 1000 Da [9]. Previous studies have found differential metabolites between PD patients and healthy controls, where the main perturbed pathways are related to the metabolism of lipids, energy (tricarboxylic acid cycle, glycolysis, acylcarnitine), fatty acids, bile acids, polyamine, and amino acids [2, 3, 16].

Non-target high-resolution mass spectrometry (NT-HRMS) coupled with liquid chromatography (LC) has become an established approach for the broad screening of thousands of chemicals in complex samples. NT-HRMS is a sensitive, fast, and accurate technology capable of detecting very small amounts of analyte (ppb or even lower concentrations, depending on the matrix) [17]. LC-HRMS can work with lower sample amount and purity than other analytical techniques such as NMR, while it is not limited to just volatile chemicals as for gas chromatography (GC). The LC-HRMS sample preparation is relatively straightforward for the matrices of interest in exposomics.

NT-HRMS post-acquisition data processing is used to identify “targets” (known chemicals), “suspects” (potential chemicals of interest based on prior knowledge), and the relevant “unknowns” or “non-targets.” However, confidence in the HRMS identifications can differ between studies and substances and are thus often reported using a 5-level identification confidence scheme [18]. This ranges from level 1, the ideal situation, where the proposed structure is confirmed through the measurement of a reference standard, through to level 5, an exact mass of interest but no chemical identity. The identification of “unknown” chemicals using NT-HRMS is a real challenge due to the limited number of reference spectra and authentic standards. In response to this need, open databases have emerged to assist in identification efforts, including the MassBank of North America (MoNA) [19] for mass spectral matching, as well as the Human Metabolome Database [20] for metabolomics or PubChemLite for Exposomics [21], a smaller subset of the 111-million-entry open chemistry database PubChem [22]. PubChem also added functionality to cross-link relationships between chemicals and diseases [23], which could be leveraged for exposomics.

NT-HRMS of complex matrices in metabolomics studies generates huge amounts of data, especially when the number of samples required for sufficient statistical relevance is high. Therefore, computational tools capable of processing such data amounts are needed to enable the identification of important chemicals in the data. Since other publications describing at least 85 metabolomics software resources, packages, and tools appeared recently [24–26], only the open-source approaches used in the present work are described here. MS-DIAL is a software pipeline that allows the identification and quantification of small molecules by mass spectral deconvolution [27]. The software platform patRoon [28, 29] provides comprehensive, fully tailored non-target analysis workflows in R, harmonizing many available tools and approaches. MetFrag supports compound identification including in silico fragmentation, matching experimental data with mass spectrometry databases like MoNA and additional metadata along with other features [30, 31]. Finally, MetaboAnalyst is a web interface (also available as an R package) that performs data processing, analysis, and interpretation in both targeted and non-targeted studies [32, 33].

In the present work, plasma and fecal samples from PD and healthy control patients were analyzed by LC-HRMS in order to identify endogenous chemicals that could be modified in PD in comparison to healthy controls, but also exogenous chemicals or those derived from the metabolism of exogenous agents such as pharmaceuticals or pesticides. The fecal samples offered additional glimpses into possible connections to the microbiome. An analytical and cheminformatics workflow was optimized, starting from the databases mentioned above but expanding into more disease-specific suspect lists (detailed below), showcasing different data analysis approaches to investigate unknown chemicals in the biological samples in an exposomics context. The biological interpretations presented in the following sections should be treated cautiously due to the limited number of samples available for this study, and will require validation with a larger cohort of samples in a future study.

## Materials and methods

### Sample preparation

A total of 22 plasma and 19 feces samples were collected as described previously [10, 34], aliquoted, and stored at − 80 °C until analysis. The collection and analysis of PD samples (plasma and feces) were part of the MiBiPa project at the University of Luxembourg in collaboration with the Paracelsus-Elena-Klinik in Kassel [35]. Demographic and medication data of the patients can be found in Table S1.

#### Plasma

Blood samples were collected from 11 PD patients and 11 healthy volunteers (Ctrl). The preparation protocol described below was adapted from the LCSB metabolomics platform protocol and previously reported methods [36–38]. Briefly, plasma samples were thawed on ice. Next, 50 µL of plasma was mixed with 50 µL of MilliQ H_2_O, vortexed, and centrifuged (12,000 g) for 6 min at 4 °C. The clear supernatant was transferred into Eppendorf tubes. Four hundred microliters of methanol (MeOH) was added to each sample to precipitate the proteins. Then, samples were incubated for 15 min at − 20 °C, then centrifuged again (12,000 g, 4 °C for 5 min). The remaining supernatant was filtered with a Phree Phospholipid Removal plate. Afterwards, samples were evaporated to dryness in a rotary vacuum evaporator and reconstituted with 0.1% formic acid (FA) in MilliQ H_2_O and MeOH (90/10, v/v). Next, samples were spiked with four internal standards (IS). Finally, samples were filtered with PHENEX-RC 4-mm syringe filters into LC vials with micro inserts and screw caps and injected in the LC-HRMS instrument. Further details are given in the supplementary information (SI) (Sect. 1).

Additionally, pooled quality control (QC) samples were prepared: 10 µL of each extracted plasma sample was taken and mixed in an Eppendorf tube. Afterwards, 16 small aliquots were prepared and put into different LC vials. The QC samples were analyzed prior to the first sample injection, after every 5 injections and at the end of the experiment, following guidelines from Broadhurst et al. [39]. The QC samples were used to filter the number of features that were considered during the data analysis, as explained in the following section. The IS were spiked in all samples to check the instrument performance, but were not included in the data analysis. To test the system suitability, blank extraction samples were prepared using 50 µL of water instead of plasma.

#### Feces

**Fig. 2:**
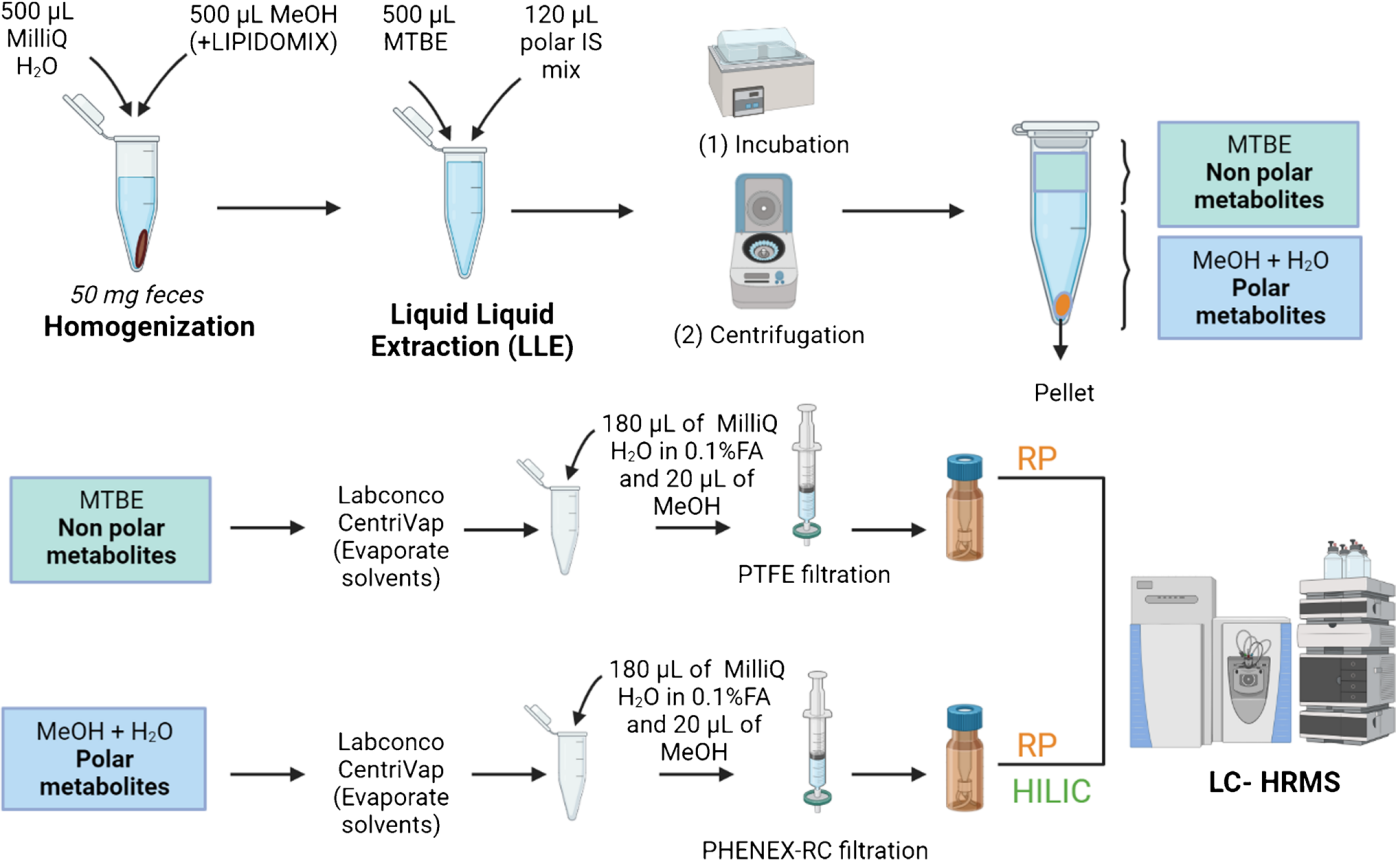
Feces sample extraction protocol. IS: internal standard, RP: reversed phase, HILIC: hydrophilic interaction liquid chromatography, MTBE: methyl tertiary-butyl ether, PTFE: polytetrafluorethylene

Feces samples were collected from 19 volunteers (10 PD patients and 9 Ctrl). The extraction protocol included the non-polar and polar fraction of the feces and was adapted from internal protocols with some modifications [40–45]. Detailed information can be found in the SI (Sect. 2). Briefly: first, samples were thawed on ice and homogenized with MilliQ H_2_O:MeOH, 1:10:10 (w/v/v) for 30 s, 6000 rpm, and 4 °C. To check the quality of the measurements, MeOH contained the SPLASH® LIPIDOMIX® Mass Spectrometry Standard. Then, a liquid–liquid extraction (LLE) step was performed to separate the polar and non-polar analytes by adding methyl tert-butyl ether (MTBE) containing a non-polar IS. In addition, 120 µL of a polar mix of IS was added to each sample. Next, samples were vortexed (10 s) and incubated for 15 min, 4 °C, and 1400 rpm. Afterwards, feces samples were centrifuged (5 min, 4 °C, 1400 rpm). The upper organic phase was transferred to new Eppendorf tubes for the extraction of the non-polar analytes, while the lower phase was moved to different Eppendorf tubes for the analysis of the polar chemicals present in the feces samples. Then, all the Eppendorf tubes (with polar and non-polar fractions) were transferred to the Labconco CentriVap to evaporate the solvents to dryness (− 4 °C for 24–48 h). Afterwards, samples were reconstituted with 180 µL of MilliQ H_2_O in 0.1% FA and 20 µL of MeOH (the non-polar fraction was spiked with an additional IS). Then, samples were filtered with a Phenex PTFE syringe filter (non-polar fraction) or PHENEX-RC 4-mm syringe filters (polar fraction). Finally, the samples were injected into the LC-HRMS system. The polar fraction was injected in both RP and HILIC, the non-polar fraction just in RP. QC and extract blank samples were prepared as described above for the plasma samples. Figure 2 shows the feces extraction protocol schematically. The spiked IS were used to test the instrument performance, as mentioned above.

### LC-HRMS analysis

Liquid chromatography analysis was performed on a Thermo Scientific Accela LC system. The injection volume was set at 5 µL. Two LC methods, RP (non-polar to slightly polar compounds) and HILIC (more polar compounds), were used in order to separate a broad range of chemicals [46]. For the RP method, an Acquity Ultra Performance Liquid Chromatography (UPLC) BEH C_18_ column (dimensions: 1.7 μm, 2.1 × 150 mm) from Waters was connected to the LC system with an optimized temperature of 35 °C. The flow was set to 0.20 mL/min using 0.1% FA in MilliQ H_2_O (A) and MeOH (B) as the mobile phases (MP). The MP gradient was the following: 90A/10B at 0 min, 90/10 at 2 min, 0/100 at 15 min, 0/100 at 20 min, 90/10 at 21 min, and 90/10 at 30 min. A SeQuant® ZIC-pHILIC 5-µm polymer (dimensions: 150 × 2.1 mm) was used to carry out the HILIC method. MP A was 20 mM ammonium acetate (pH = 9) in LC–MS-grade acetonitrile, while MP B was 20 mM ammonium acetate (pH = 9) in MilliQ H_2_O. The gradient program was as follows: 10A/90B at 0 min, 10/90 at 1.5 min, 80/20 at 16 min, 80/20 at 18 min, 10/90 at 20 min, and 10/90 at 33 min. The column temperature was 50 °C and the flow rate 0.2 mL/min.

The Q Exactive™ HF (Thermo Scientific) mass spectrometer was used in both positive ( +) and negative ( −) electrospray ionization (ESI) modes. Thus, four methods were performed for each individual sample: RP in ESI ( +) and ESI ( −) and HILIC in ESI ( +) and ESI ( −). The following full MS/data-dependent (dd) MS^2^ settings were used: resolution (MS1 = 120,000 for the RP method and MS1 = 60,000 for the HILIC method, at *m*/*z* 200), automatic gain control (AGC) target (1.0 × 10^6^), maximum injection time (IT) 70 ms, and scan range (*m*/*z* = 60–900). On the other hand, for the dd-MS^2^/dd-SIM (data-dependent selected ion monitoring), the following settings were used: resolution (MS2 = 30,000 at *m*/*z* 200), AGC target (5.0 × 10^5^), maximum IT 70 ms, loop count (10 for RP and 5 for HILIC), Top N (10 in RP and 5 in HILIC), isolation window (1.0 Da), and (N)CE (30 for RP and 20 for HILIC). Lastly, the following dd settings were used: minimum AGC target (8.0 × 10^3^, intensity threshold (1.1 × 10^5^), apex trigger (4 to 6 s), exclude isotopes (On), and dynamic exclusion (10 s). The instrument was calibrated and optimized every time an analysis was performed using manufacturer settings to ensure consistent performance throughout the whole study.

### Data processing

**Fig. 3:**
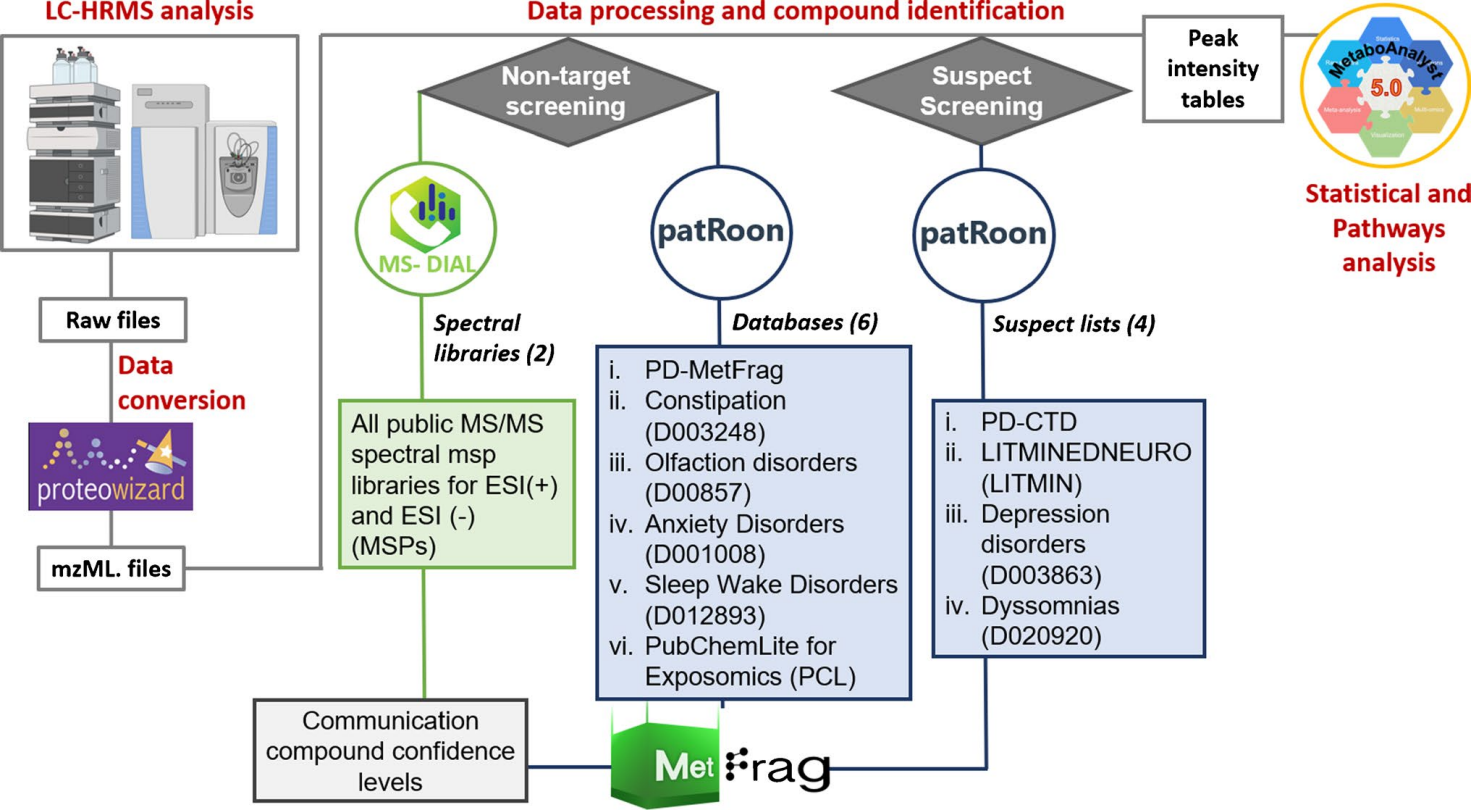
Data analysis workflow. First, samples were injected in the LC-HRMS system and raw files were obtained and converted to mzML. These files were used to perform the non-target and suspect screening. Codes used for the identification of each list are shown in brackets. In the last step, MetaboAnalyst 5.0 was used to perform the statistics and pathway analysis

Raw files were converted to mzML format using MSConvertGUI (Version 3.0.20331.3768aa6e9 64-bit), from ProteoWizard [47]. The converted files were analyzed with MS-DIAL [27] (version 4.70), for non-target screening, and patRoon [28] (version 1.2.0), for both suspect and non-target screening, yielding features/intensity tables. For each feature in each QC sample, mean, standard deviation (SD), and relative standard deviation (RSD) values were calculated. Only features with RSD < 50% in QC samples were considered for further analysis. All selected features that passed the quality control were annotated using a level-based identification confidence scheme [18]. The greatest focus was given on annotating the chemicals by library spectral match (level 2a) using two values—the *individualMoNAscore* (patRoon) and the *Dot product* (MS-DIAL). These indicate the quality of match between the samples and spectra in the respective MS libraries (for both, a higher score indicates a better match). Since three different approaches were used to analyze the data (non-target screening with both MS-DIAL and patRoon, plus suspect screening with patRoon—each providing different information), the three different sets of criteria used to annotate the features with all respective levels are described in Table 1. Peak intensity tables of the annotated features derived from patRoon and MS-DIAL were then uploaded to MetaboAnalyst 5.0 for statistical and pathways analyses. The data analysis workflow showing all steps (explained in the following sections) is given in Fig. 3.

**Table 1:** Identification confidence level system used to annotate the chemicals in each different cheminformatics approach employed in this work. Not all levels or sublevels were employed in all approaches (the “-” indicates that this level/sublevel was not considered in this study)

	**patRoon** **Suspect screening**	**patRoon** **Non-target screening**	**MS-DIAL** **Non-target screening**
Level 2a	MoNAScore > 0.9 One candidate only	MoNAScore > 0.9 One candidate only	≥ 3 ion fragments matching Dot product 70–100 Fragment presence 50–100
Level 2b	-	-	≥ 3 ion fragments matching Dot product 70–100 Fragment presence 50–100 Structure unknown in library
Level 3a	MoNAScore > 0.4	MoNAScore 0.7–0.9	≥ 3 ion fragments matching Dot product 50–70 Fragment presence 50–100
Level 3b	> 3 fragments match If < 3 fragments, all match	MoNAScore 0.4–0.7	< 3 ion fragments matching Dot product 50–100 Fragment presence 50–100
Level 3c	annSimComp > 0.7	-	< 3 ion fragments matching Dot product 50–100 Fragment presence 50–100 Structure unknown in library
Level 4a	Top ranked formula annSimForm ≥ 0.7 isoScore ≥ 0.5 annSimFomp and isoScore 0.2 higher than next candidate	-	Dot product < 50 and/or fragment presence < 50
Level 4b	Top ranked formula isoScore > 0.9 and > 0.2 higher than the next candidate	-	-
Level 5	Unknown mass of interest	-	-

*MoNAScore* individualMoNAscore, *annSimComp* annotation MS/MS similarity

#### MS-DIAL non-target screening

All public MS/MS spectral MSP-formatted libraries (MSPs) for both positive and negative ionization modes were used for data processing and compound identification, which included 13,303 unique compounds for MS/MS positive and 12,879 unique compounds for MS/MS negative at the time this work was performed (April 2021). Features with QC RSD > 50% or without MS/MS assigned, m/z match or MS/MS match were discarded. The MS-DIAL parameters used are summarized in Table S2.

#### patRoon non-target and suspect screening

The non-target screening workflow was as follows: Firstly, features were extracted and grouped with *XCMS3* [48, 49], then a data clean-up step (*filter()* function) was performed to increase the quality of the dataset. Next, relevant MS data was extracted using the *mzR* algorithms [50]. Molecular formulas were calculated using *GenForm* [29, 51, 52], and compound identification was performed with *MetFrag* (MetFrag2.4.5-CL jar file) [31] and the *PubChemLite for Exposomics* (PCL) database [53]. Additionally, different databases and suspect lists were used, as described below in the “10” section.

For suspect screening, the same parameters were used as above, with the additional incorporation of the “*ScreenSuspects*” and “*annotateSuspects*” functions to screen the chemicals from the selected suspect lists. All R scripts used for the data analysis are available on the ECI GitLab repository [54].

#### Disease-specific databases and suspect lists

Several disease-specific and open-access databases and suspect lists were used with patRoon and will be described as follows. The LITMINEDNEURO (LITMIN) [55] dataset of 1243 chemicals associated with neurotoxicity [17] was downloaded from CompTox [55–57], and the PCL database was downloaded from Zenodo [58]. Further databases and suspect lists (depending on the size of the outcomes) for specific neurological diseases were also developed for non-target and suspect screening, respectively, with patRoon. The creation and PD curation of these lists is documented on GitLab [54], while all newly created lists are available in Zenodo [59]. A list of 296 chemicals associated to PD in the Comparative Toxicogenomic Database (CTD) [47] was extracted via the CTD integration within PubChem (PD-CTD). Details about these three resources (PCL, LITMIN, PD-CTD) are given in Table 2.

**Table 2:** Summary of the chemicals associated with PCL, LITMIN, and PD-CTD resources (full names in the table)

Name	Code	Type of list	CIDs
PubChemLite for Exposomics	PCL	Database	371,663
LITMINEDNEURO	LITMIN	Suspect list	1243
Chemicals associated with PD in the Comparative Toxicogenomic Database (CTD)	PD-CTD	Suspect list	296

Furthermore, PubChem functionality relating Medical Subject Headings (MeSH) information on certain disease endpoints to chemicals in PubChem was explored to create additional lists and databases of chemicals associated with PD and related disorders [23]. Firstly, chemicals co-occurring with MeSH terms (D000544, D003704, D006816, D010300, D010302, D019636, D020734, and D020961) were merged into a single MetFrag database (PD-MetFrag). Information about MeSH codes and the number of entries is given in Table 3. Finally, a further six lists were developed related to characteristic disorders in the PD pre-motor stage: constipation, sleep and olfaction disorders, depression, and anxiety (for details, see Table 3). These disorders were selected as they usually appear in the earlier stages of the disease, before the motor features are detected, which is a critical time point for diagnosis and specific treatment.

**Table 3:** Summary of all lists (database or suspect list), developed by MeSH code, to study chemicals associated to PD (and related disorders). The “type of list” column indicates if the table was small enough for being treated as a suspect list (for suspect screening) or whether they were treated as a database (for non-target screening). The last column indicates the code used to identify each list in this manuscript. Note that the CIDs related to the 8 first MeSH were de-duplicated and merged to create a single database (PD-MetFrag). Complete tables can be downloaded in Zenodo [59]

Disorder	MeSH	CIDs	Type of list	Code
Parkinson disease	D010300	21,303	Database	PD-MetFrag
Parkinson disease, secondary	D010302			
Parkinsonian disorders	D020734			
Lewy body disease	D020961			
Alzheimer disease	D000544			
Dementia	D003704			
Huntington disease	D006816			
Neurodegenerative diseases	D019636			
Constipation	D003248	3943	Database	D003248
Olfaction disorders	D000857	688	Database	D000857
Anxiety disorders	D001008	8433	Database	D001008
Sleep wake disorders	D012893	2781	Database	D012893
Depression	D003863	363	Suspect list	D003863
Dyssomnias	D020920	59	Suspect list	D020920

Overall, five specific databases related to neurological diseases and disorders (PD-MetFrag, D003248, D00857, D001008, D012893) were created, along with three suspect lists for depression, dyssomnias, and PD (PD-CTD) to complement PCL, LITMIN, and the MSPs.

### Statistical and pathways analysis

Peak intensity tables (in comma-separated values (CSV) file format) that were obtained following compound annotation with patRoon and MS-DIAL were uploaded to MetaboAnalyst 5.0 for statistical analysis. The preliminary processing steps included data filtration [using the interquartile range (IQR) option], normalization by sum, and then Pareto Scaling. For univariate analysis, fold changes (FC) and *T* test *p* values were calculated. Multivariate exploratory analysis was performed using principal component analysis (PCA), orthogonal projections to latent structures discriminant analysis (OPLS-DA), and variable importance on projection (VIP) scores to determine which compounds were best or worst at distinguishing between the PD and Ctrl groups (VIP > 1).

Features were considered statistically relevant when *T* test *p* values < 0.1, FC > 2 (higher levels in the PD group) or FC < 0.5 (higher levels in the Ctrl group), and VIP score > 1. All features had to meet these 3 conditions to be considered statistically significant in this study. The false discovery rate (FDR) *t* test was not used here as this would have resulted in no significant differences—likely due to the relatively low number of samples available for use. The statistical analysis was performed separately for each different database or suspect list investigated in this manuscript, then compared and discussed in the next section.

Finally, based on the altered compounds observed between the studied groups, pathway analysis was performed to select the metabolic pathways potentially correlated to PD on the features selected as statistically relevant in PD using the KEGG *Homo sapiens* pathway library. Out of the three types of input data, compound name, HMDB ID, and KEGG ID, only the first one was selected for the analysis. Duplicates were removed prior to the analysis.

## Results and discussion

### Compound annotation

The total number of features identified in each different approach (before and after QC) is summarized in Table S3; the plasma and feces annotations are shown in Table S4 and Table S5, respectively.

**Fig. 4:**
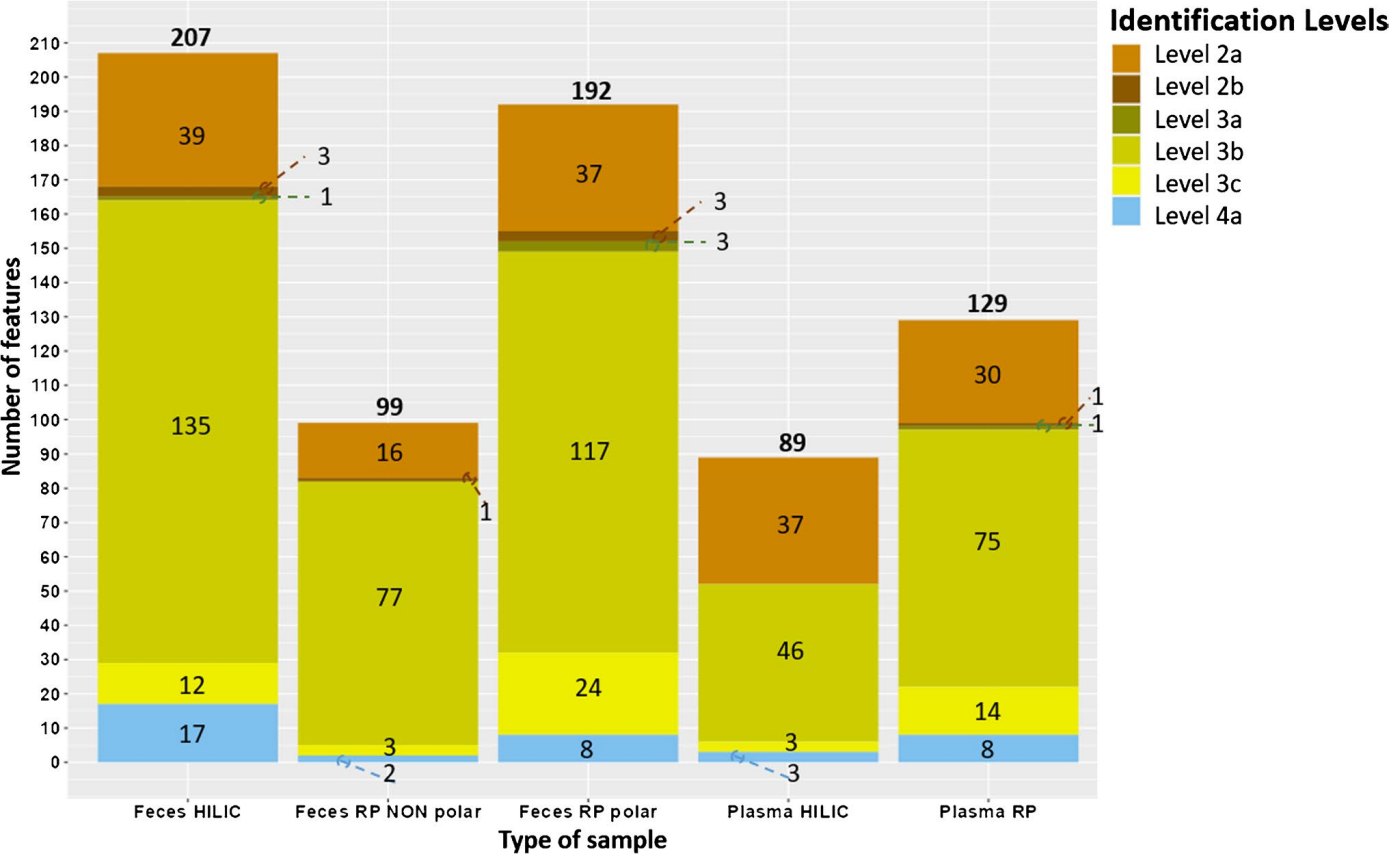
Total number of features identified using MS-DIAL software and the public MSPs for ESI ( +) and ESI ( −). Note that features identified in ESI ( +) and ESI ( −) were combined (duplicates were not removed); more details can be found in Tables S4–S5. The number found at the top of each bar plot indicates the total number of features identified in all levels

Figure 4 shows the identification levels of the total number of features that were tentatively identified with MS-DIAL MSPs. The relevant features identified via MS-DIAL are described further in the following sections. While several level 2a results were achieved (very good spectral match, ≥ 30 per sample except for non-polar feces), the majority of features were identified as level 3b, meaning good dot and fragment scores, but fewer than three ion fragment matching with the reference spectra. This could be either due to insufficient collision energy for some spectra (e.g., a higher collision energy could result in more fragment information and maybe a higher number of level 2a annotations), or due to the fact that an Orbitrap mass spectrometer was used in this study, while the MSPs contain many Q-TOF spectra, which are somewhat less comparable. Additionally, some level 2b features were found, which meant a very good spectral match to a reproducible feature with unknown structure in the library.

For the non-target screening using patRoon (Fig. S2), most features passed the QC and were annotated using the PCL dataset [53] (except for feces HILIC), followed by PD-MetFrag. Most of the features were annotated as level 2a (i.e., with very good MoNAScore), and thus a very good library match.

For the suspect screening, out of the 4 lists screened, most of the chemicals were found with the LITMIN and PD-CTD suspect lists (Fig. S3). Level 2a annotations came mainly from the LITMIN list (which is significantly bigger than the PD-CTD list, with 1243 entries vs. 296 entries). A comparison of level 2a features in plasma RP and feces RP-polar revealed a low overlap between LITMIN and the other lists (Table S6). However, the majority of features (248 in plasma and 371 in feces; see Table S4 and Table S5, respectively) were only annotated at level 5, with insufficient information available to annotate those chemicals further.

Different criteria were used to annotate the features (summarized in Table 1), according to the data available from the diverse software and approaches used in this study, as explained above. Hence, the direct comparison of level 2a features (chemicals with higher identification confidence) across MS-DIAL and patRoon should be done with a degree of caution, since there is not exactly the same information available for the same feature when using one software or another. Thus, the higher number of level 2a features identified by MS-DIAL could be explained by the different criteria employed for the level communication. Additionally, an update to the MoNA library integrated in MetFrag could result in a higher number of level 2a features obtained via patRoon. It is important to note that in contrast to non-target approaches, patRoon suspect screening automatically calculates the identification levels (not yet present for non-target screening), making the communication of confidence easier and more reproducible across laboratories. Finally, the different criteria used for confidence level communication should be considered when comparing results across the different software employed by a single research group but also across different research groups.

### Enhancing chemical detection analytically

**Fig. 5:**
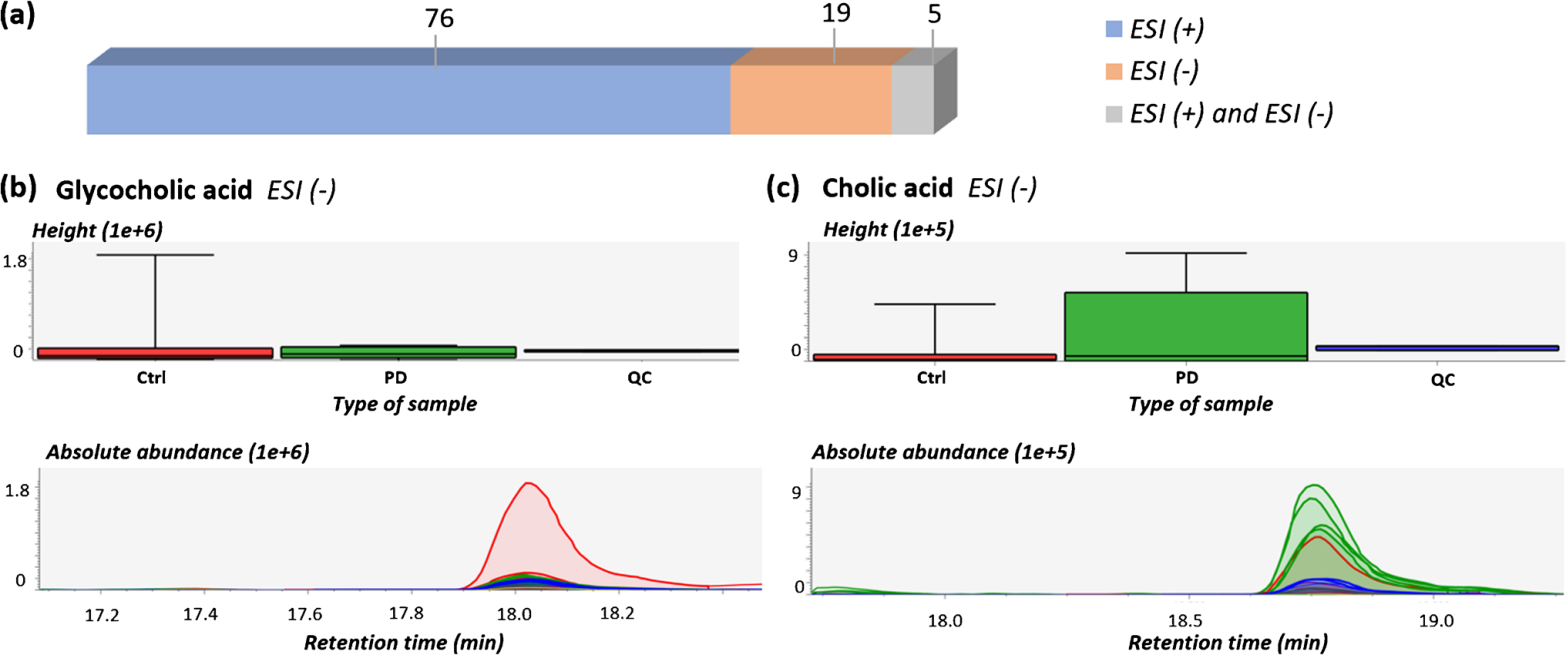
(a) Bar plot showing the overlapping between ESI ( +) and ESI (* −*) annotated features in plasma RP (obtained by MS-DIAL MSPs). InChIKeys were used to compare both approaches; thus, level 2b and 3c features are excluded as they are “unknown” and the InChIKeys are not available. (b) Glycocholic acid bar chart plot (top), based on the average peak height, and extracted ion chromatogram (EIC), bottom. m/z = 464.3015. (c) Cholic acid bar chart plot based on the average peak height (top) and EIC (bottom). m/z = 407.2804. The green color refers to the PD group; red is associated to Ctrl and blue to QC samples

Samples were measured in both ESI ( +) and ESI ( −), with a low overlap of chemicals between the modes in all approaches, as can be observed in Table S3. While most features were identified in ESI ( +) using all approaches, the use of ESI ( −) was important, allowing the identification of biological features that may play an important role in the pathogenesis of PD, such as bile acids (e.g., cholic acid) since they can be better ionized in ESI ( −) mode. Figure 5 exemplifies this with the plasma RP annotations (using MS-DIAL MPSs), where only five features overlap between ESI ( +) and ESI ( −). Two of the 19 unique features identified by ESI ( −) were found with reasonable confidence levels: glycocholic acid (level 2a) and cholic acid (level 3b).

**Fig. 6:**
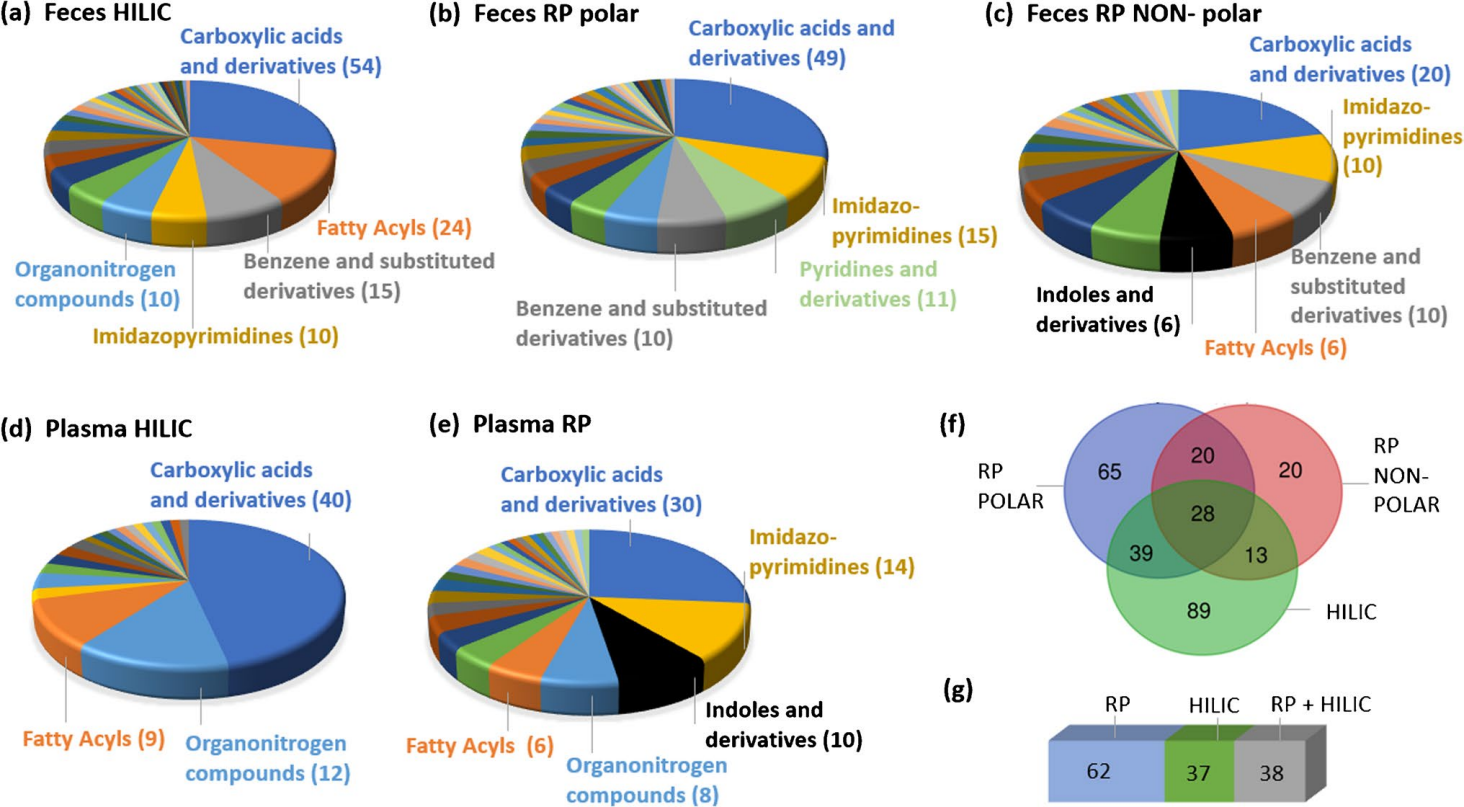
Classification of chemicals identified in (a) feces HILIC, (b) feces RP-polar, (c) feces RP-NON-POLAR, (d) plasma HILIC, and (e) plasma RP. (f) and (g) show the overlapping across RP and HILIC in feces and plasma, respectively. Note that the features detected in positive and negative ionization modes have been combined per matrix and LC mode, for simplicity. The classification of each of the tentatively identified chemicals can be found in tables S4 (for plasma) and S5 (for feces), in the column labeled “class.” Chemical classification was performed with ClassyFire [60]

In addition, relevant chemicals were found with each of the different chromatographic settings, i.e., with HILIC and RP. Figure 6 shows the various classes of chemicals annotated in RP and HILIC (plasma and feces matrices), using MS-DIAL MSPs. Figure 6 (f) and (g) show the overlap between both chromatographic methods. Most features were uniquely identified with one of the two approaches (RP or HILIC), although some overlap was observed. Both methods allowed the separation of carboxylic acids and derivatives, although more of this class of compounds was separated with HILIC in both feces and plasma samples. On the other hand, more imidazopyrimidines were separated with the RP column. Thus, the combination of two chromatographic methodologies (RP and HILIC) allowed the separation of a broad range of chemicals, from highly polar (HILIC column) to weakly polar and non-polar (RP column).

Overall, the development of an analytical pipeline that combines ESI ( +) and ESI ( −) ionization modes, as well as RP and HILIC chromatographic methods, improves the chemical coverage in biological samples, which is particularly interesting for non-target studies. However, implementing more approaches also results in more data and consequently more complex data analysis—thus, there is not an ideal analytical method, and many variables should be considered (e.g., relevant chemicals, time, budget) when selecting the analytical method.

### Overlap between databases and suspect lists

**Fig. 7:**
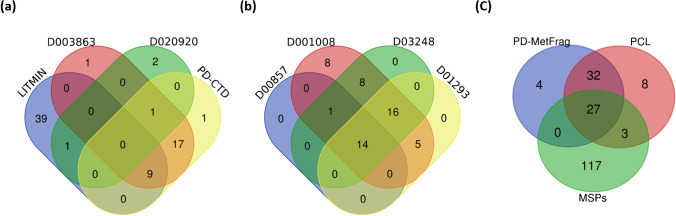
Venn diagram of features annotated in feces RP polar. Only level 2 and 3 annotations were considered in these plots. (a) Suspect list comparison. (b) Comparison of the smallest databases used in patRoon. (c) MS-DIAL MSPs, PCL (patRoon), and PD-MetFrag (patRoon) comparison. Plots were performed with the Draw Venn Diagram tool from the University of Ghent [61]

Since many databases and suspect lists were used in this study (see “2” and Tables 2 and 3), the overlap of features annotated using the different lists is explored in Fig. 7. This compares (a) suspect screening with the non-target screening approaches (b and c) in feces RP polar, which was the matrix with the highest number of features found. Figure 7 includes only level 2 and 3 features, compared via InChIKey across the lists, with duplicates removed prior to plotting. Of the 184 level 2 and 3 features identified by the MSPs (Fig. 7 (c)), the features annotated as level 2b and level 3c were discarded as the InChIKey information is unavailable. Thus, only 157 features were considered, corresponding to 147 the unique features included in the plot (10 duplicates were removed). More information about the duplicate features discarded in each of the databases/suspect lists can be found in the SI, Table S7.

For the suspect lists (Fig. 7 (a)), the LITMIN list showed a higher number of unique features (39) while the other lists only provided between one and two unique features. Nevertheless, a further 22 features were identified with the other lists together that were not in LITMIN. Out of the 22 features, 19 were obtained with the PD-CTD list. These features included valine, an essential amino acid with increased levels in the PD group (see next section). As indicated in the plot, 17 features were commonly identified with the D003863 and PD-CTD, but not with the other lists, resulting in a similar effectiveness of these two lists. The D020920 list revealed the lowest number of features which could be explained by the low number of CIDs present in the list (59). Thus, the combination of different suspect lists (LITMIN + PD-CTD or LITMIN + D00857) enhanced the identification of features that might be related to PD in comparison when a single suspect list is considered.

Figure 7 (b) represents the comparison of the 4 smallest databases studied with patRoon. The D001008 list (which has a higher number of CIDs compared to the other three lists, details in Table 3) showed eight unique features, while the other lists did not provide any unique features. Unlike the suspect screening lists (Fig. 7 (a)), many of the entries detected in these databases overlapped, with 14 features found in all databases and another 16 in three of the four (D001008, D03248, and D01293).

Finally, Fig. 7 (c) compares the two largest databases investigated using patRoon (PCL and PD-MetFrag) with the MSPs screened in MS-DIAL. A large number of features were obtained with MS-DIAL MSPs, as commented previously. Moreover, 117 of them were unique when compared with PCL and PD-MetFrag databases. A further 44 features were identified using PD-MetFrag and PCL (32 of them overlapping) that were not in the MSPs. Additionally, as discussed further in the next section, some of these features were specifically associated to PD or related disorders.

### Selection of significant features to classify PD patients

#### Non-target screening

A total of 48 features (31 unique) were identified as statistically significant from MS-DIAL and patRoon results, considering the two MSPs and the six databases; see Table S8. Most of the features were identified in ESI ( +) (only three were found in ESI ( −)) and in feces samples (only four features were statistically relevant in plasma). MS-DIAL provided a higher number of unique statistically relevant features (20 out of 31). Six of the 31 features were annotated at level 2a; of these, three were with MS-DIAL (alanine betaine, 3-(3-hydroxyphenyl) propionic acid (3-HPPA), and choline) and three with patRoon (L-valine, nicotinamide, and isonicotinic acid). The last two features were identified using more than one database, as indicated in Table 4*.* All of these level 2a features were found in feces samples. L-Valine was only identified as statistically relevant via the D00857 database, although this chemical is also present in PCL. The L-valine VIP value obtained through PCL screening was less than 1 (VIP = 0.7783); therefore, it did not pass the filter to be selected as relevant in this context. This shows that incorporating more than one database could provide different insights of PD metabolome and exposome, but also that the number of candidates in the database/suspect list can influence the number of features annotated and thus the statistical outcomes. While Table 4 summarizes only the statistically relevant features annotated as level 2a, Table S8 contains all the statistically relevant chemicals, identified in plasma and feces by non-target approaches, from level 2a to level 4. The first column indicates whether the chemical was found in feces or plasma. Higher levels of p-coumaraldehyde (level 3b) and vanillin (level 4) were found in plasma of PD patients compared to the Ctrl group, while higher levels of ethylparaben and 1-(3-(trifluoromethyl)phenyl)piperazine (TFMPP) were found in the PD feces samples, both identified as level 3b. More examples of statistically relevant features identified in these matrices can be found in Table S8.

**Table 4:** Features identified in feces samples with a high confidence level (annotated as level 2a) and with statistically significant differences between PD and Ctrl groups (*p* value < 0.1, FC < 0.5 or FC > 2, VIP score > 1). No level 2a features were identified as statistically relevant in plasma samples. Table S8 contains all statistically relevant chemicals identified in plasma and feces samples by non-target approaches (from level 2a to level 4)

Separation mode	Tentative candidate	Adduct	Fold change	*T* test *p* value	VIP score	m/z	RT (min)	Formula	Database/library
HILIC	Alanine betaine	[M + H] +	0.2875	0.0388	2.0537	132.1016	4.35	C_6_H_13_NO_2_	MSPs
RP (non-polar fraction)	3-HPPA	[M-H]-	0.1459	0.0693	1.7102	165.0547	13.14	C_9_H_10_O_3_	MSPs
RP (polar fraction)	Choline	[M] +	2.0994	0.0856	2.1461	104.1067	1.88	C_5_H_14_NO	MSPs
RP (polar fraction)	L-Valine	[M + H] +	2.2627	0.0165	2.2821	118.0861	2.15	C_5_H_11_NO_2_	D00857
HILIC	Nicotinamide	[M + H] +	0.2333	0.0922	1.6385	123.0550	2.47	C_6_H_6_N_2_O	D00857, D003248, D001008, D012893, PD-MetFrag, PCL
RP (non-polar fraction)	Isonicotinic acid	[M + H] +	2.8013	0.0416	2.1909	124.0389	2.45	C_6_H_5_NO_2_	PD-MetFrag, PCL

**Fig. 8:**
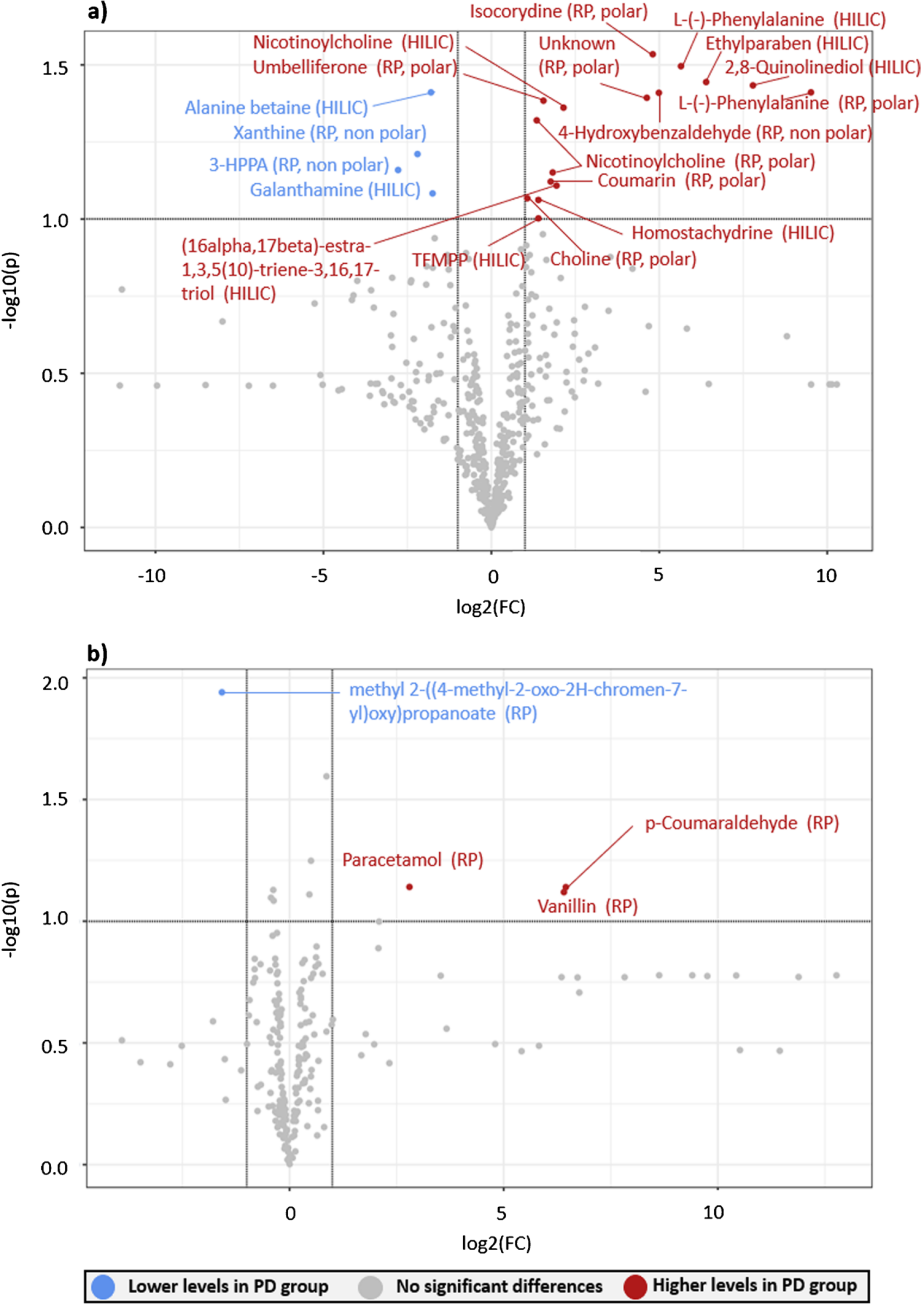
Important features detected in feces (a) and plasma (b) selected by volcano plot with FC threshold (*x*) 2 and *t* tests threshold (*y*) 0.1. Only features identified by the MSPs are included in the plot. The red circles represent features above the threshold. Note both fold changes and *p* values are log transformed. Increasing distance from 0.0 indicates increasing significance

Figure 8 represents a volcano plot containing all the statistically relevant features, derived from the MSPs, in plasma and feces samples. Each dot represents a different feature, with the chromatographic method (RP, HILIC) shown in brackets. This clearly illustrates which features are found with statistically higher levels in the PD group (red) or lower (blue) compared to the Ctrl group. Only the features identified by the MSPs are included in the plot, since most of the statistically relevant features were found there, as discussed above.

**Fig. 9:**
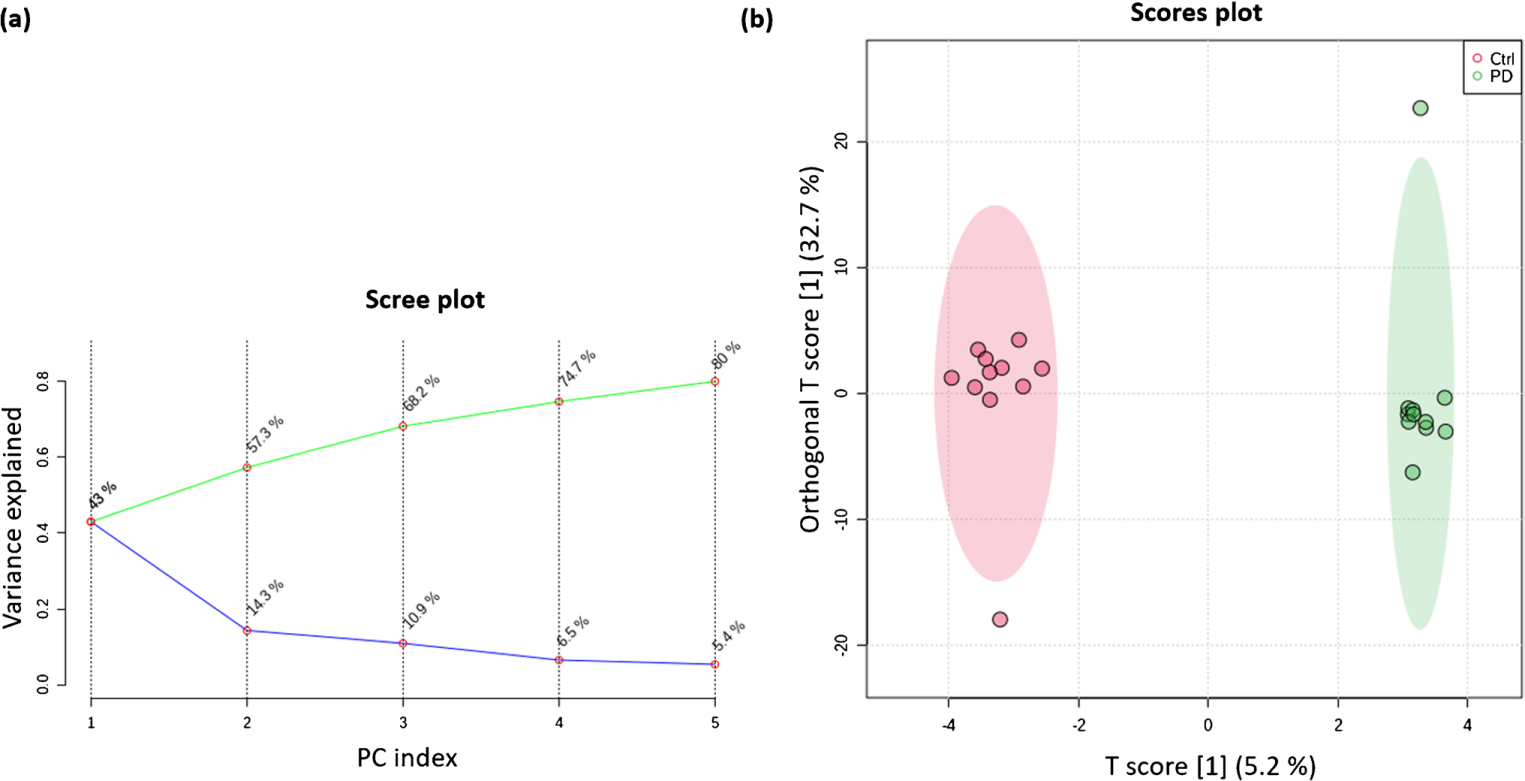
Multivariate statistics of all chemicals identified in plasma samples by MS-DIAL. (a) PCA scree plot showing the variance explained by 5 PCs. (b) OPLS-DA score plot

In addition to the univariate statistics, multivariate analysis (unsupervised; PCA and supervised; OPLS-DA) was performed for each different approach to evaluate the separation between groups. In the plasma samples, all statistically significant features (4) were obtained with MS-DIAL and some separation was observed on the PCA, shown in Fig. 9 (a). As can be seen, 80% of the variance is explained by adding 5 PCs. Figure 9 (b) shows the OPLS-DA analysis, where the separation between PD (green) and Ctrl (red) groups can be observed.

Regarding the feces multivariate analysis, PCA and OPLS-DA were very similar across the different databases and libraries. For example, PC1 explains 18.4%, 16.3%, and 16.0% of variability when we used MS-DIAL, PD-MetFrag, and PCL annotations respectively (Fig. S4–S6).

#### Suspect screening

As performed for the non-target approaches, the suspect screening results for both plasma and feces samples were analyzed statistically. In total, 74 statistically significant features were identified using the PD-CTD, LITMIN, and D003863 suspect lists: two features were classified as level 2a, 17 were classified as level 3c, only one feature was classified as level 4b, and 54 were annotated as level 5. No statistically relevant features were identified with the D020920 suspect list. Detailed information can be found in the SI (Table S9).

Out of the level 2a and level 3c features, there were 2, 8, and 5 unique features identified in the D003863, LITMIN, and PD-CTD lists, respectively. Thus, as explained in previous sections, PD-CTD and LITMIN lists are good options to screen potential biomarkers in PD. Moreover, the multivariate statistics of these last two lists (Fig. S7–S8) show similar results; PC1 explains 16.4% and 17.6% of the variance, for LITMIN and PD-CTD, respectively.

Levodopa and nicotinamide (Table 5) were classified as level 2a, but only the last one was highlighted as statistically relevant in non-target screening approaches.

**Table 5:** Features identified in plasma and feces samples with a high confidence level (annotated as level 2a) and with statistically significant differences between PD and Ctrl groups (*p* value < 0.1, FC < 0.5 or FC > 2, VIP score > 1) among all suspect lists studied. Detailed information can be found in SI (Table S9)

Sample	Tentative candidate	Separation mode	Adduct	Fold change	*T* test *p* value	VIP score	Formula	m/z	RT (min)	Suspect list
Plasma	Levodopa	RP	[M + H] +	9.8904	0.0525	1.7131	C_9_H_11_NO_4_	198.0756	2.38	PD-CTD
Feces	Nicotinamide	HILIC	[M + H] +	0.1621	0.0621	1.8863	C_6_H_6_N_2_O	123.0550	2.44	LITMIN

Figure S9 shows the important features detected in plasma and feces samples with the PD-CTD suspect list. Most of the significant features are “unknown” (annotated as Level 5 by patRoon), and it would be interesting to further investigate the identity of these features, since they could have an impact in the development of the disease. The statistically relevant but unknown features identified in this work will be incorporated into a data-dependent acquisition list for future efforts with a larger cohort of samples to validate the results presented in this work and gain additional information to help identify the unknown chemicals presented here.

In summary, multivariate analysis showed differences across groups via non-target and suspect screening. Both approaches helped discover relevant features in PD, one being a focused set of chemicals (suspect screening) and the other one trying to broadly identify as many chemicals as possible (non-target). Moreover, both approaches could be complementary, e.g., levodopa was found via the non-target databases; nevertheless, it was only highlighted as statistically relevant when using the data from the PD-CTD suspect list.

### Relevance of the statistically significant features found in PD

Some statistically relevant chemicals explored with MetaboAnalyst and derived from non-target (Table S8) and suspect screening approaches (Table S9) will be discussed in the following paragraphs. If a chemical is not present in Table S8 or S9, this means that no statistically significant differences across groups were found. In most cases, statistically significant differences between PD and Ctrl groups were found only in feces, but not in the plasma samples. Table S4 indicates whether the chemical was identified in plasma samples. If the chemical is present in Table S4 but not in Table S8 or S9, it means that it was identified in the plasma samples but without statistically significant differences between PD and Ctrl groups.

Significantly lower levels of 3-(3-hydroxyphenyl) propionic acid (3-HPPA) were found in feces samples of PD patients compared to Ctrl (Table 4). 3-HPPA was not identified in the plasma samples analyzed in this study. The chemical 3-HPPA is generated by gut microbiota fermentation of dietary polyphenols (e.g., coffee, tea, fruits, and vegetables) and drugs (such as levodopa) [62]. Previous studies reported that 3-HPPA might attenuate α-Syn aggregation [63]. This result would agree with the hypothesis explained in previous sections, where microbiome dysbiosis in PD could modify the levels of some gut metabolites, affecting the α-Syn aggregation, and thus the PD progression.

On the other hand, nicotinamide, the active form of vitamin B_3_, was found decreased in PD feces samples. This metabolite was also identified in the plasma samples, although no significant differences were found between the two groups. Nicotinamide can be directly produced by the gut microbiota, showing anti-inflammatory and antioxidant activities, hence playing a neuroprotective role. Lower levels of nicotinamide would increase the oxidative stress, being implicated in PD pathogenesis [11]. Moreover, a recent study suggests that vitamin B_3_ supplements in PD patients could maintain or improve the symptoms, therefore ameliorating the quality of life of these patients [64].

Alanine betaine, an alanine derivative, was detected at lower levels in the feces of PD patients compared to Ctrl. Gut microbiota impacts on their production which could explain the variability observed in the disease group [65]. Alanine betaine was only identified in feces, not in plasma samples. Previous studies have found phenylalanine pathways to be altered in PD; nevertheless, to the best of our knowledge, none of them highlight this specific metabolite.

L-Valine, a hydrophobic essential amino acid, was found significantly increased in the PD feces. By contrast, significantly lower levels of valine were found in the plasma samples of the PD group compared with the control. Alterations in amino acid levels of PD patients are common and have been reported in different types of samples [2, 16]. Choline, which was also increased in PD patients’ feces samples, is an essential nutrient for humans. This compound was also detected in 4 samples, but no significant differences were observed between the groups under study. While humans can produce it in small quantities, most of it comes from the diet (e.g., eggs, meat, and fish). Choline is metabolized by the gut microbiota to trimethylamine (TMA), which is absorbed by the host and converted into trimethylamine-N-oxide (TMAO) in the liver. Higher levels of TMA and TMAO in the body are related to inflammatory disease and cardiovascular diseases among others [66]. TMAO was detected in both plasma and feces samples with high confidence, but without statistically significant differences between the PD and Ctrl groups. Detailed information about plasma and feces annotations can be found in Tables S3 and S4, respectively.

As shown in Table S8, higher levels of TFMPP were found in the PD feces compared to the Ctrl group but it was not detected in the plasma samples. This chemical is classified as an environmental contaminant and psychotropic drug. Neurotoxic effects of TFMPP and its derivatives (2-TFMPP and 4-TFMPP) have been reported previously [67]. In addition, higher levels of ethylparaben were detected in the PD feces compared to the Ctrl group, but it was not detected in the plasma samples. Parabens, such as ethylparaben, are used as a preservative in food, personal care products, and pharmaceuticals, and are continuously released into the environment. Concerns regarding the safety of parabens in humans and the environment are increasing, with toxicity reported in *Caenorhabditis elegans* models [68]. It does not appear that any studies focused on the possible relationship between TFMPP and/or paraben exposure and PD development have been published to date.

Overall, a higher number of statistically significant features were observed in feces samples (25 unique features), when compared with plasma (four unique features), via non-target approaches. Since most PD metabolomics studies so far have focused mainly on blood and CSF matrices [2, 15, 16], this study highlights the importance of feces samples in PD metabolomics studies, where the microbiome dysbiosis might have a great impact in the pathogenesis of the disease. Therefore, the identification of altered chemicals in feces will help better understand the role of dysbiosis in the development of PD.

Finally, the new cheminformatics approach presented in this manuscript enabled the identification of endogenous metabolites that could be related to PD (e.g., nicotinamide) but also exogenous chemicals, such as TFMPP, which may help gain a better understanding of the environmental contribution to the PD development. It is important to note, as mentioned in previous sections, that due to the limited number of samples analyzed in this study, the results shown here must be validated with a larger cohort of samples in a future study.

### Pathways analysis

**Fig. 10:**
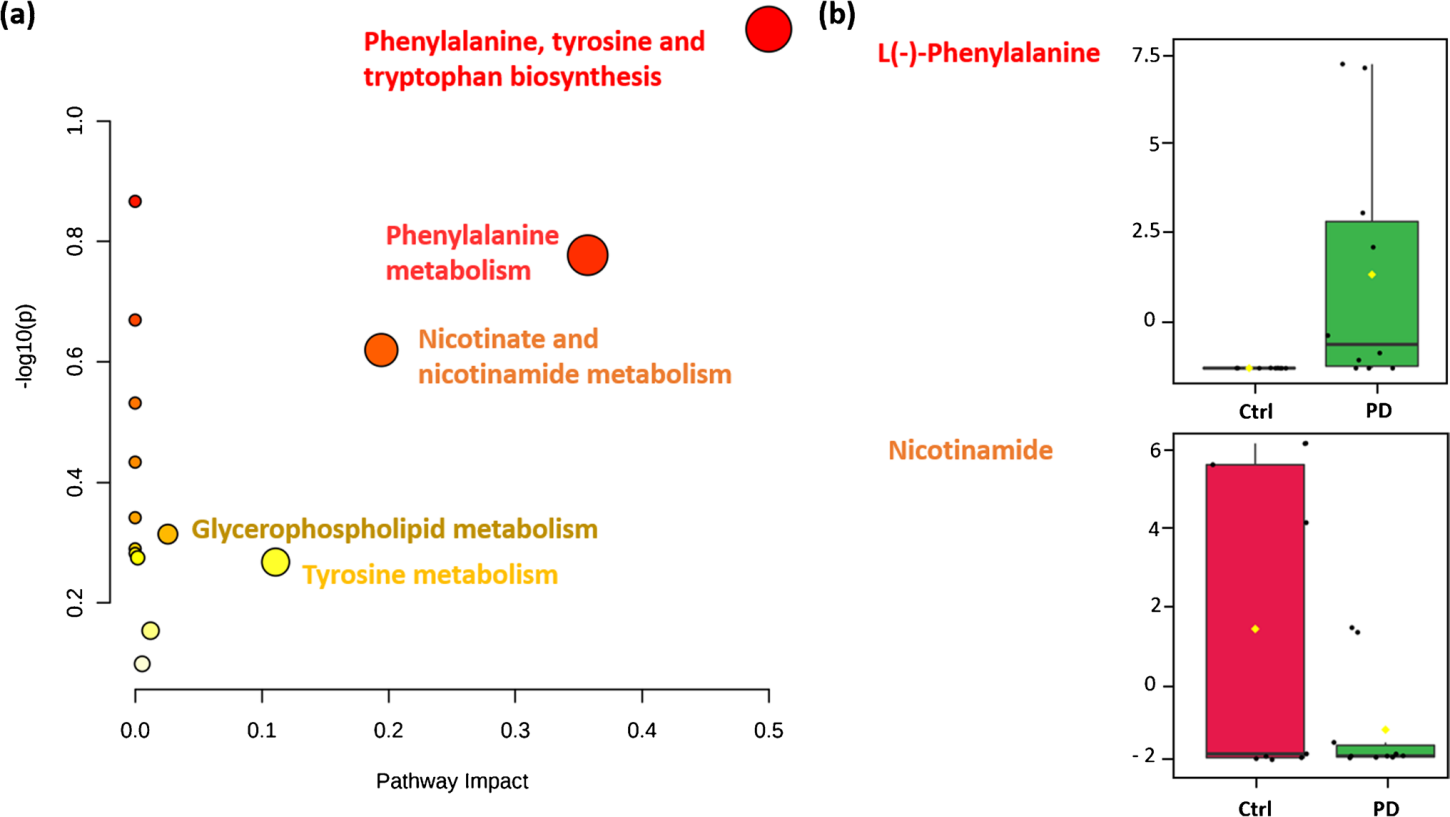
(a) Pathways analysis of the statistically relevant chemicals found in plasma and feces samples of PD patients by non-target and suspect screening approaches. The greater the color intensity, the greater the relevance in PD. Moreover, the larger the dot and the greater the coordinate values, the more important the pathway. (b) Box plots of selected metabolites with significantly different concentrations in feces between PD (green) and Ctrl (red) groups. Fold changes and *p* values are provided in Tables S8–S9

Figure 10 shows a visual representation of the main metabolomic pathways that are altered among the PD patients studied in this work. The pathway analysis was conducted based on the 46 unique chemicals observed as altered in PD by non-target screening and the 13 unique features (level 2a and level 3c) identified by suspect screening. For that purpose, duplicated names between both approaches were removed; then, the 43 compound names (one of the only three input options) were uploaded to MetaboAnalyst 5.0; the input table is given in SI, Table S10. It would be interesting if future versions of MetaboAnalyst could consider additional identifiers such as SMILES, InChIKeys, or PubChem CIDs in their options, as well as an extension of the pathway analysis/integrated databases to also include exogenous chemicals.

The pathways analysis shows that the phenylalanine, tyrosine, and tryptophan biosynthesis pathways are closely related to PD. Phenylalanine, an essential amino acid, is converted to tyrosine, and used in the biosynthesis of dopamine and norepinephrine neurotransmitters. Tryptophan is another essential amino acid, precursor of the neurotransmitter serotonin. Other pathways that might be altered are nicotinate and nicotinamide metabolism, which are related to the oxidative stress, known to play an important role in the pathogenesis of the disease, as explained in previous sections. These results are in line with previous studies [16, 69].

## Conclusions

This study provides a new methodological framework to analyze unknown chemicals in PD biological samples in a non-target manner. The combination of different analytical methods, RP and HILIC, ESI ( +) and ESI ( −), enhances the separation and identification of features from the analyzed samples, providing new and complementary insights into the PD metabolome and exposome. While using a single database or suspect list to analyze chemical data has been a common approach applied so far in metabolomics studies, this work explores the use of specific databases and suspect lists with chemicals that may be related to the initial stages of the disease, thus trying to identify molecular hallmarks that could help in an earlier identification of PD. These results show that non-target screening with larger databases can provide better results, compared with smaller ones, since this allowed the identification of a larger number of relevant features (MSPs of MS-DIAL, or PD-MetFrag and PCL with patRoon), which then provided larger lists for more robust statistical analyses. PCL and PD-MetFrag provided almost the same number of level 2a features, suggesting that both would be good options for non-target screening with patRoon to answer PD-related exposomics questions. On the other hand, the screening of all MSPs with MS-DIAL is an excellent alternative to perform non-target screening, as a large number of level 2a features were obtained that had low overlap with other approaches.

Suspect screening approaches remain a good alternative if the study is focused on identifying a certain number of chemicals. Additionally, since the communication of confidence levels using patRoon is automatic for suspect screening, this is more reproducible across laboratories. In this work, the LITMIN and PD-CTD lists yielded the higher number of level 2a features.

Most of the relevant features were found in feces samples, highlighting the importance of considering this matrix in metabolomics studies of PD. This also implies that gut microbiome dysbiosis might have a major impact on the development of PD, and may be responsible for the altered levels of chemicals found in the PD biological samples. Since some chemicals derived from the environmental exposure (TFMPP and ethylparaben) were found statistically higher in the PD group, environmental exposures should be taken into consideration in further non-target studies of PD.

Further efforts are ongoing to apply the optimized cheminformatics approach, described in this manuscript, on a larger sample size, with the aim of identifying more statistically relevant features. Additionally, reference standards are being acquired to confirm the identity of chemicals of interest and to potentially allow for quantification in future measurements.

## Supplementary Information

The Supplementary Information available online includes tables S1-S10 (Excel format) and a Word file containing 4 sections (S): S1. Protocol plasma preparation, S2. Protocol feces preparation, S3. Compound annotation and S4. Statistics.

Below is the link to the electronic supplementary material.

Supplementary file1 (XLSX 346 KB)

Supplementary file2 (DOCX 1086 KB)

## Data Availability

All suspect lists and databases developed in this work are freely available on Zenodo (10.5281/zenodo.6382057). The PubChemLite for Exposomics database used in this work is available for download on Zenodo (10.5281/zenodo.4183801), while updated versions of PubChemLite are available at 10.5281/zenodo.5995885. The LITMINEDNEURO dataset is online in CompTox (https://comptox.epa.gov/dashboard/chemical-lists/LITMINEDNEURO). The MSP spectral libraries used with MS-DIAL can be found online (http://prime.psc.riken.jp/compms/msdial/main.html#MSP).

## Abbreviations

( −): Negative
( +): Positive
3-HPPA: 3-(3-Hydroxyphenyl)propionic acid
AGC: Automatic gain control
CID: Compound identification number
CNS: Nervous central system
CSF: Cerebrospinal fluid
CSV: Comma-separated values
CTD: Comparative Toxicogenomic Database
Ctrl: Healthy control group
CUDA: 12-[[(Cyclohexylamino)carbonyl]amino]dodecanoic acid
dd: Data dependent
ddSIM: Data-dependent selected ion monitoring
EIC: Extracted ion chromatogram
ESI: Electrospray ionization
FA: Formic acid
FC: Fold change
GC: Gas chromatography
GI: Gastrointestinal
HILIC: Hydrophilic interaction liquid chromatography
HRMS: High-resolution mass spectrometry
IQR: Interquartile range
IS: Internal standards
IT: Injection time
LC: Liquid chromatography
LCSB: Luxembourg Centre for Systems Biomedicine
LITMIN: LITMINEDNEURO
TFMPP: 1-(3-(Trifluoromethyl)phenyl)piperazine
LLE: Liquid-liquid extraction
LRRK2: Leucine-Rich Repeat Kinase 2
MeOH: Methanol
MeSH: Medical Subject Headings
MGBA: Microbiota-gut-brain axis
MoNA: MassBank of North America
MoNAScore: IndividualMoNAScore
MP: Mobile phase
MSPs: MS/MS spectral msp libraries
MTBE: Methyl *tert*-butyl ether
NT: Non-target
OPLS-DA: Orthogonal Projections to Latent Structures Discriminant Analysis
PCA: Principal component analysis
PCA1: First principal component
PCL: PubChemLite for Exposomics
PD: Parkinson’s disease
PMIDs: PubMed IDs
QC: Quality control
RBD: REM-sleep behavior disorder
RP: Reversed phase
RSD: Relative standard deviation
SD: Standard deviation
SI: Supplementary information
TMA: Trimethylamine
TMAO: Trimethylamine-*N*-oxide
VIP: Variable importance on projection
α-Syn: Alpha-synuclein

## Code availability

The primary software programs used in this study, patRoon 1.2.0 (https://github.com/rickhelmus/patRoon/releases/tag/1.2.0) and MS-DIAL 4.70 (http://prime.psc.riken.jp/compms/msdial/main.html), are both open source. The code, functions, and files associated with this manuscript are provided in the ECI GitLab repository (https://gitlab.lcsb.uni.lu/begona.talavera/pd-cheminformatics-pipeline).
