# Supplementary material for: Studying the Parkinson’s disease metabolome and exposome in biological samples through different analytical and cheminformatics approaches: a pilot study
Source: Anal Bioanal Chem. 2022 Jul 13;414(25):7399–419. doi: 10.1007/s00216-022-04207-z (PMC9482909; doi:10.1007/s00216-022-04207-z)
Supplement: Supplementary file 2 — Supplementary file2 (DOCX 1086 KB) [file 216_2022_4207_MOESM2_ESM.docx]

**Supplementary Information**

**Studying the Parkinson’s disease metabolome and exposome in biological samples through different analytical and cheminformatics approaches: a pilot study**

Begoña Talavera Andújar^1*^, Dagny Aurich^1^, Velma T.E. Aho^1^, Randolph R. Singh^1,2^, Tiejun Cheng^3^, Leonid Zaslavsky^3^, Evan E. Bolton^3^, Brit Mollenhauer^4,5^, Paul Wilmes^1,6^, Emma L. Schymanski^1*^

^1^ Luxembourg Centre for Systems Biomedicine (LCSB), University of Luxembourg, Avenue du Swing 6, L-4367 Belvaux, Luxembourg

^2^ IFREMER (Institut Français de Recherche pour l’Exploitation de la Mer), Unité Contamination Chimique des Ecosystèmes Marins, Nantes, France

^3^ National Center for Biotechnology Information, National Library of Medicine, National Institutes of Health, Bethesda, MD 20894, USA

^4^ Department of Neurology, University Medical Center Göttingen, Göttingen, Germany

^5^ Paracelsus-Elena-Klinik, Kassel, Germany

^6^ Department of Life Sciences and Medicine, Faculty of Science, Technology and Medicine, University of Luxembourg, Esch-sur-Alzette, Luxembourg

*Corresponding authors: BTA: [begona.talavera@uni.lu](mailto:begona.talavera@uni.lu) and ELS: [emma.schymanski@uni.lu](mailto:emma.schymanski@uni.lu)

**Contents**

[**Section 1. Protocol plasma preparation** 2](#_Toc104824947)

[**Fig. S1**. 3](#_Toc104824948)

[**Section 2. Protocol feces preparation** 3](#_Toc104824949)

[**Section 3. Compound annotation** 5](#_Toc104824950)

[**Fig. S2.** 5](#_Toc104824951)

[**Fig. S3**.. 6](#_Toc104824952)

[**Section 4. Statistics** 7](#_Toc104824953)

[**Fig. S4.**. 7](#_Toc104824954)

[**Fig. S5.** 8](#_Toc104824956)

[**Fig. S6.**. 9](#_Toc104824957)

[**Fig. S7.**. 9](#_Toc104824958)

[**Fig. S8.** 10](#_Toc104824959)

[**Fig. S9.** 11](#_Toc104824960)

**Tables S1-S10** are available online from OwnCloud,
Link: <https://owncloud.lcsb.uni.lu/s/dKKjSzrMhUNGkf5>

Password: LCSB2022

# **Section 1. Protocol plasma preparation**

Blood samples were collected from 22 volunteers (11 PD patients and 11 Ctrl). The preparation protocol (described below) was adapted from LCSB metabolomics platform internal protocols and several studies [1–3].

A monophasic sample extraction, using MilliQ water (H_2_O) (ELGA LabWater Purelab Ultra from Labtec Services) and methanol (MeOH), was selected because its simplicity, higher reproducibility and lower variance compare to the biphasic methods, in which immiscible organic solvents are added to water [4]. First, 50 µL of plasma was mixed with 50 µL of MilliQ H_2_O, vortexed for 60 seconds, kept in ice (5 min), and centrifuged at 12, 000 g, 4 °C for six minutes. The clear supernatant was transferred into Eppendorf tubes. 400 µL of methanol (MeOH) was added into each sample to precipitate the proteins. Then, samples were vortexed and incubated for 15 minutes at -20 °C, followed by another centrifugation step (12, 000 g, 4 °C for five minutes). The remaining supernatant was filtered with a Phree Phospholipid Removal, 30 mg / well, 96-Well Plate (Phenomenex). Afterwards, samples were evaporated to dryness in a rotary vacuum evaporator (Labconco CentriVap) and reconstituted with 126 µL of 0.1% formic acid (FA) in MilliQ H_2_O and MeOH (90/10, v/v). The samples were spiked with four internal standards; α-chloralose, bromopyruvic acid, sucralose and 2-ketobutyric acid-13C_4_-D_2_ to obtain a final concentration of 1 µg/ mL. Finally, samples were filtered with PHENEX-RC 4 mm Syringe Filters into LC vials with micro inserts and screw caps and injected in the LC-HRMS instrument (**Fig. S1**).


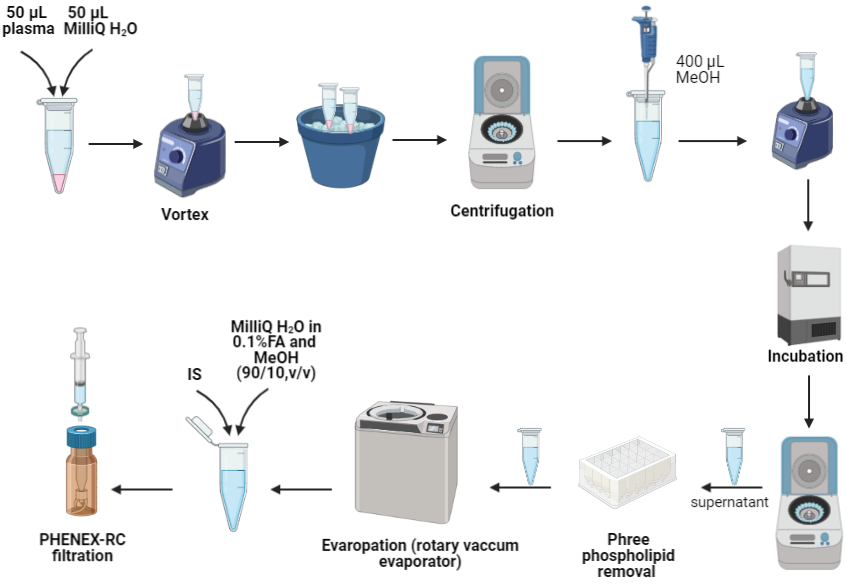


**Fig. S1**. Sample extraction procedure for plasma samples. IS: Internal Standard, MP: Mobile Phase.

# **Section 2. Protocol feces preparation**

Feces samples were collected from 19 volunteers (10 PD patients and 9 Ctrl). The extraction protocol included the non-polar and polar fraction of the feces and was adapted from internal protocols with some modifications [5–10]. A biphasic sample extraction was performed as described below.

First, samples were homogenized with MilliQ H_2_O: MeOH, 1:10:10 (w/v/v) for 30 seconds, 6,000 rpm and 4 °C. To check the quality of the measurements, MeOH contained the SPLASH® LIPIDOMIX® Mass Spectrometry Standard (dilution 1:50). Then, a liquid-liquid extraction (LLE) step was performed to separate the polar and non-polar analytes by adding 500 µL of methyl tert-butyl ether (MTBE), containing Cholesteryl ester 22:1 (Sigma-Aldrich) as non-polar IS. Moreover, 120 µL of a polar mix of IS was added to each sample to ensure the quality of the measurements. The polar mix contained 10 ng /mL of 6-chloropurine riboside, 2-chloroquinoline-3-carboxylic acid, 4-chloro-DL-phenylalanine, Nε-trifluoroacetyl-L-lysine, sucralose and caffeine-trimethyl ^13^C_3_ in MilliQ H_2_O. Samples were vortexed (10 seconds) and incubated for 15 min, 4 °C, and 1,400 rpm. Afterwards, feces samples were centrifuged (5 min, 4 °C, 1,400 rpm). Finally, the upper organic phase was transferred to new Eppendorf tubes for the extraction of the non-polar analytes. The lower phase was moved to different Eppendorf tubes for the analysis of the polar chemicals present in the feces samples.

Then, all the Eppendorf tubes (with polar and non-polar fractions) were transferred to the Labconco CentriVap to evaporate the solvents until dryness (-4 °C for 24h-48h). Afterwards, samples were reconstituted with 180 µL of MilliQ H_2_O in 0.1% FA and 20 µL of MeOH (the non-polar fraction was spiked with 10 µg/mL 12-[[(cyclohexylamino)-carbonyl]amino]-dodecanoic acid (CUDA)). Then, samples were filtered with Phenex PTFE syringe filter (non-polar fraction) or PHENEX-RC 4mm Syringe Filters (polar fraction). Finally, the samples were injected into the LC-HRMS system.

# **Section 3. Compound annotation**

**
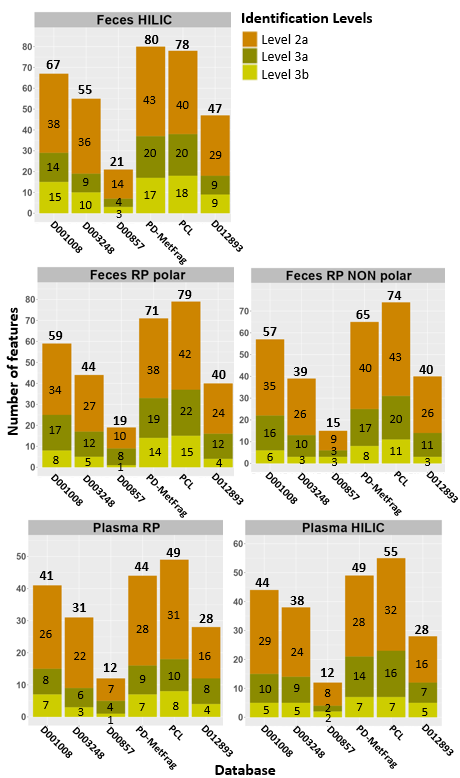
**

**Fig. S2.** Total number of features identified in the different databases tested in patRoon. After passing the QC, only features with IndividualMoNASCore >0.4 were considered and annotated. Note that for each database features identified in ESI (+) and ESI (-) were summed (duplicates were not removed). The number indicated above each bar chart is the result of the combination of all the features identified at each level for each database.


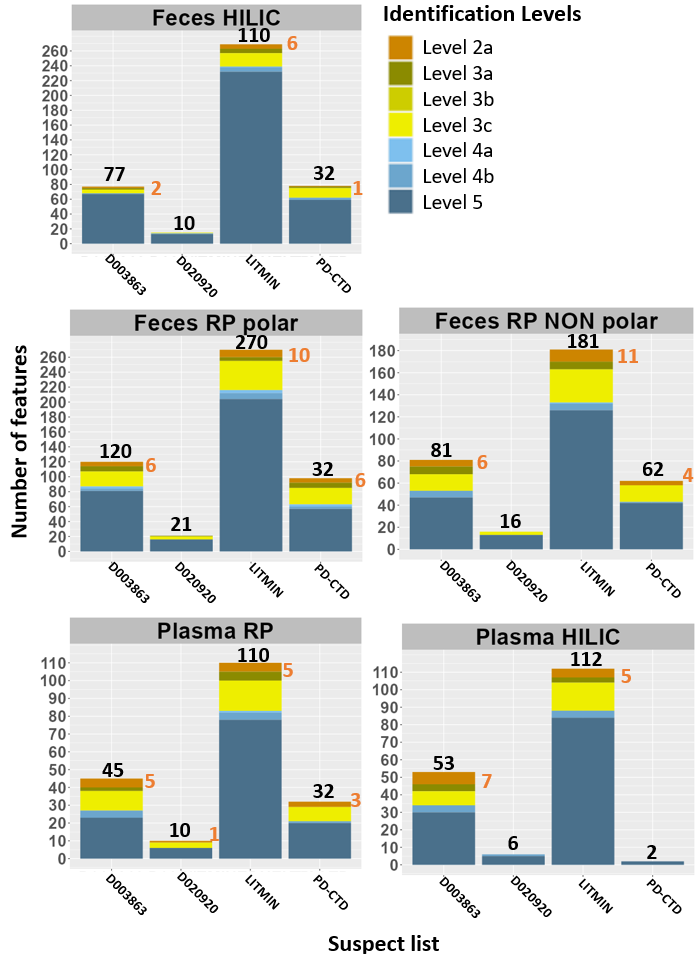


**Fig. S3**. Number of features identified by suspect screening in each sample and separation mode (RP or HILIC). Note that for each suspect list features identified in ESI (+) and ESI (-) were combined (duplicates were not removed). The number indicated above each bar chart is the result of the sum of all the features identified at each level for each suspect list. The orange number on the right side of each bar indicates the number of Level 2a annotations (no number means that there are not Level 2a features). The criteria for the feature identification levels were the same as those described in the patRoon handbook [11].

# **Section 4. Statistics**


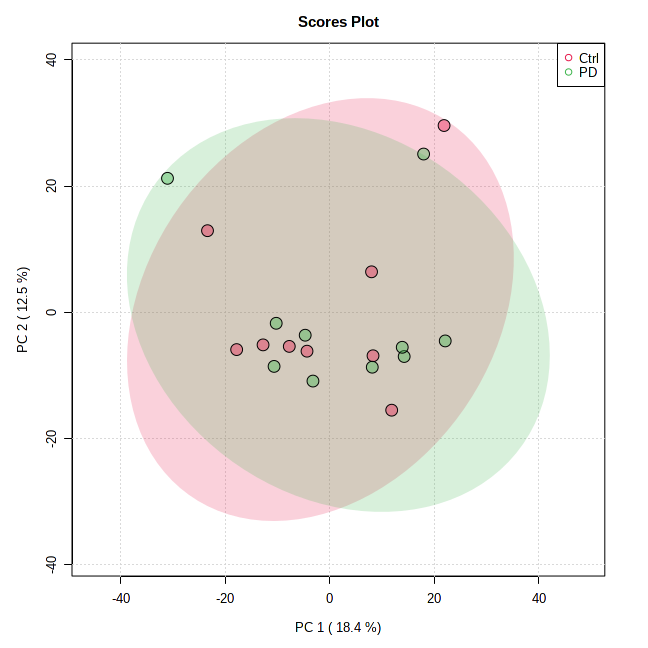


**Fig. S4.** PCA analysis of all chemicals identified in feces samples by MS-DIAL.


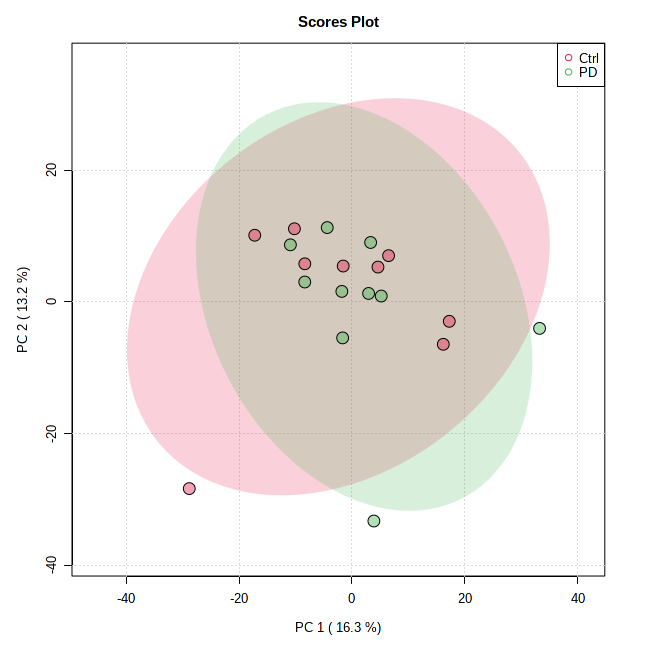


**Fig. S5.** PCA analysis of all chemicals identified in feces samples by patRoon and PD-MetFrag list.


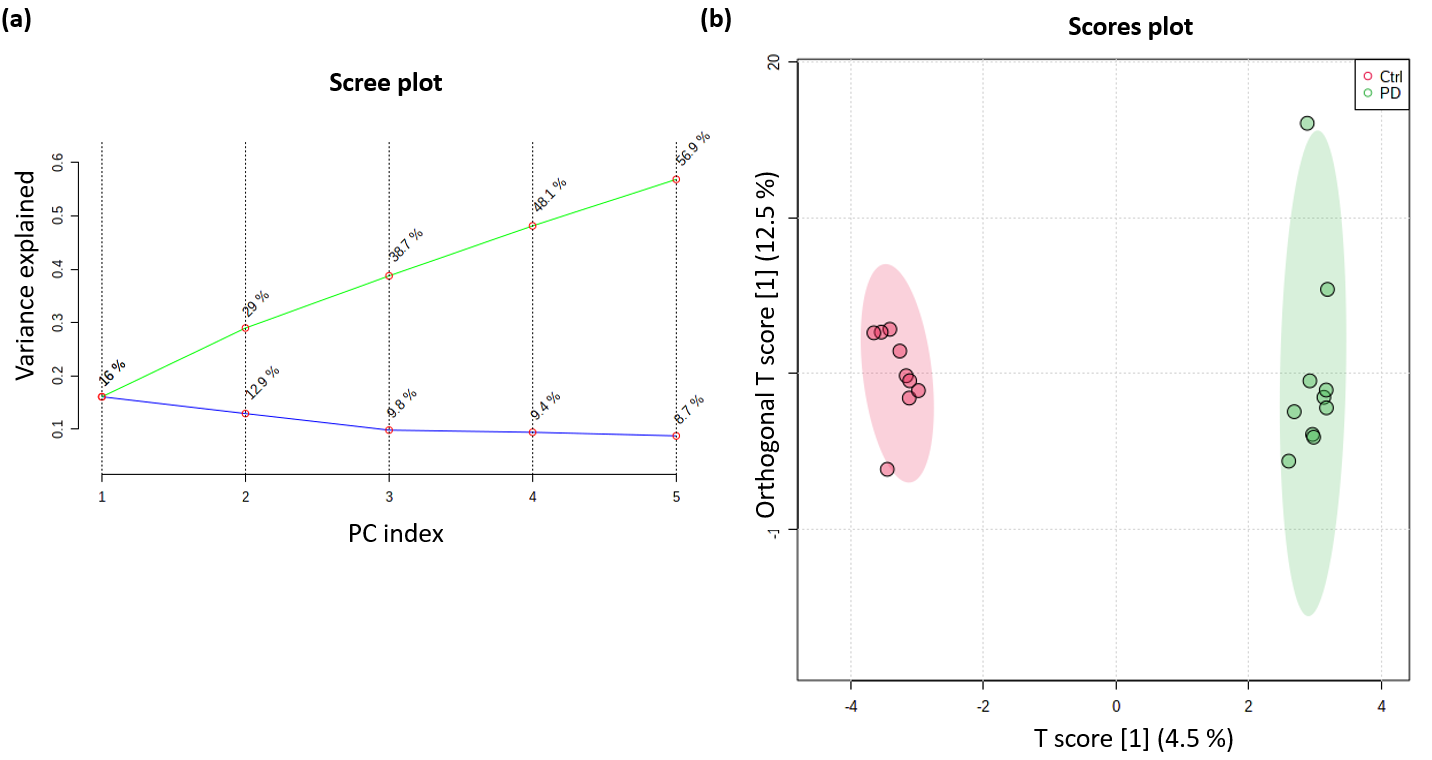


**Fig. S6.** Multivariate statistics of all chemicals identified in feces samples by patRoon non-target and PCL list. (a) PCA scree plot showing the variance explained by 5 PCs. (b) OPLS-DA score plot showing separation across PD (green) and Ctrl (red) groups.

**
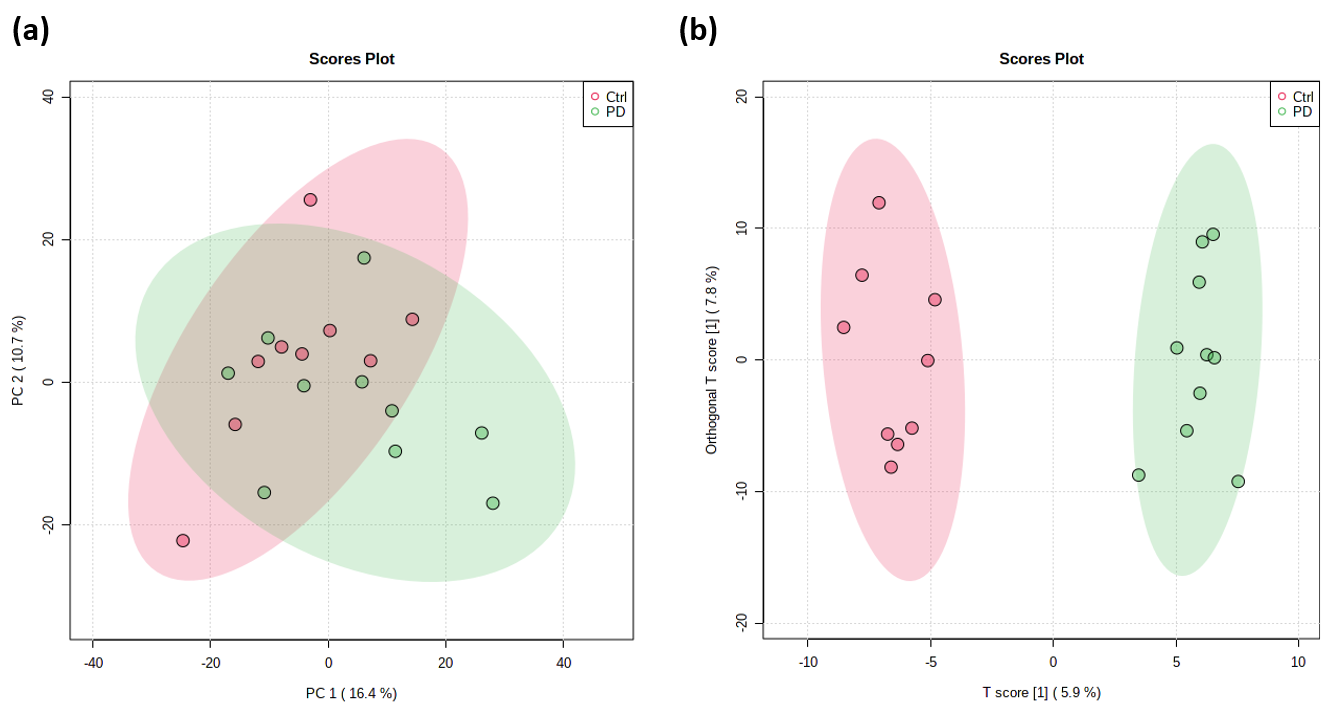
**

## ***Fig. S7.*** *(a) PCA analysis between PD (green) and Ctrl (red) groups. (b) OPLS-DA analysis maximize the separation between groups. These plots were built after analyzing the fecal data with the LITMIN list.*

**
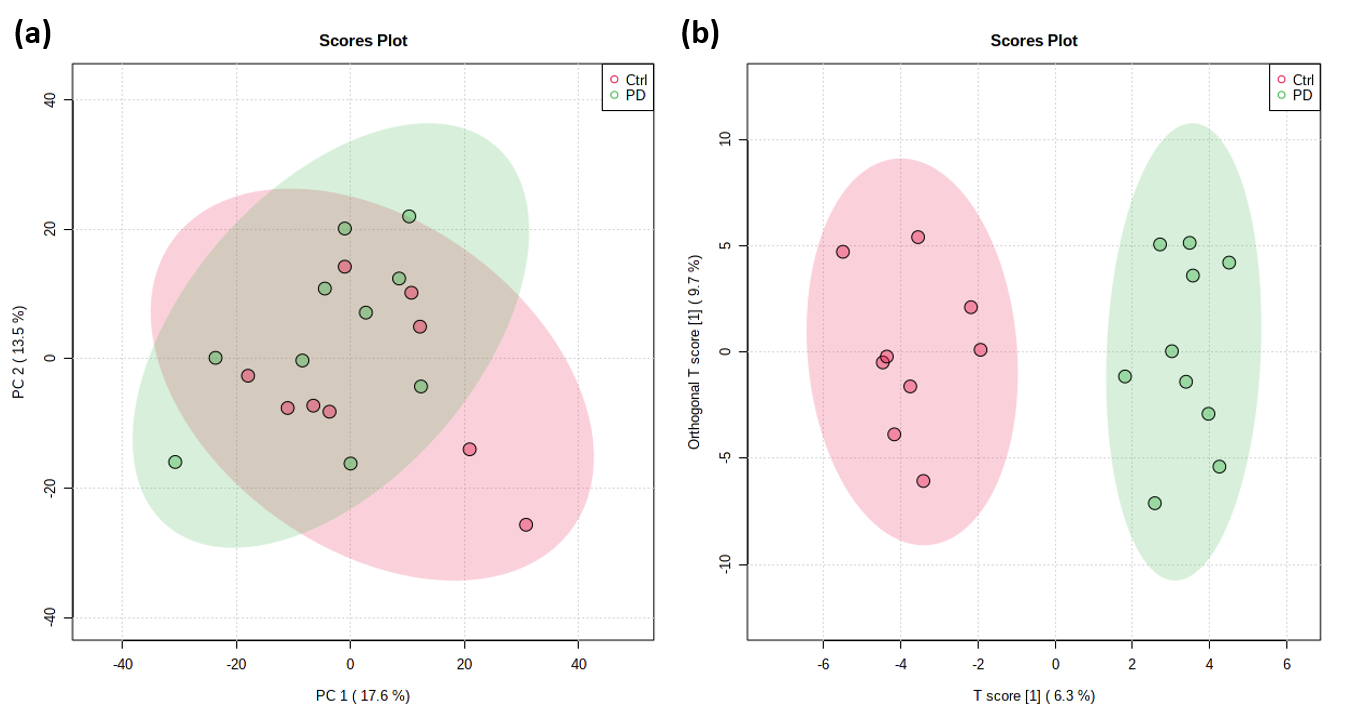
**

**Fig. S8.** (a) PCA analysis between PD (green) and Ctrl (red) groups. (b) OPLS-DA analysis maximize the separation between groups. These plots were built after analyzing the fecal data with the PD-CTD list.

**Fig. S9**. represents a volcano plot, which illustrates the statistically relevant features in plasma and feces, derived from the analysis with the PD-CTD list. Each dot represents a different feature, with the chromatographic method shown in brackets. As can be seen, most of the chemicals were unknown (annotated as Level 5) so the *m/z* was provided instead of the chemical name. In the plasma samples plot (b) you could find two dots identified as “melevodopa/ methyldopa” meaning that we did not have enough information to know which feature is (both were annotated as Level 3c). More information is available in the SI, **Table S9**.


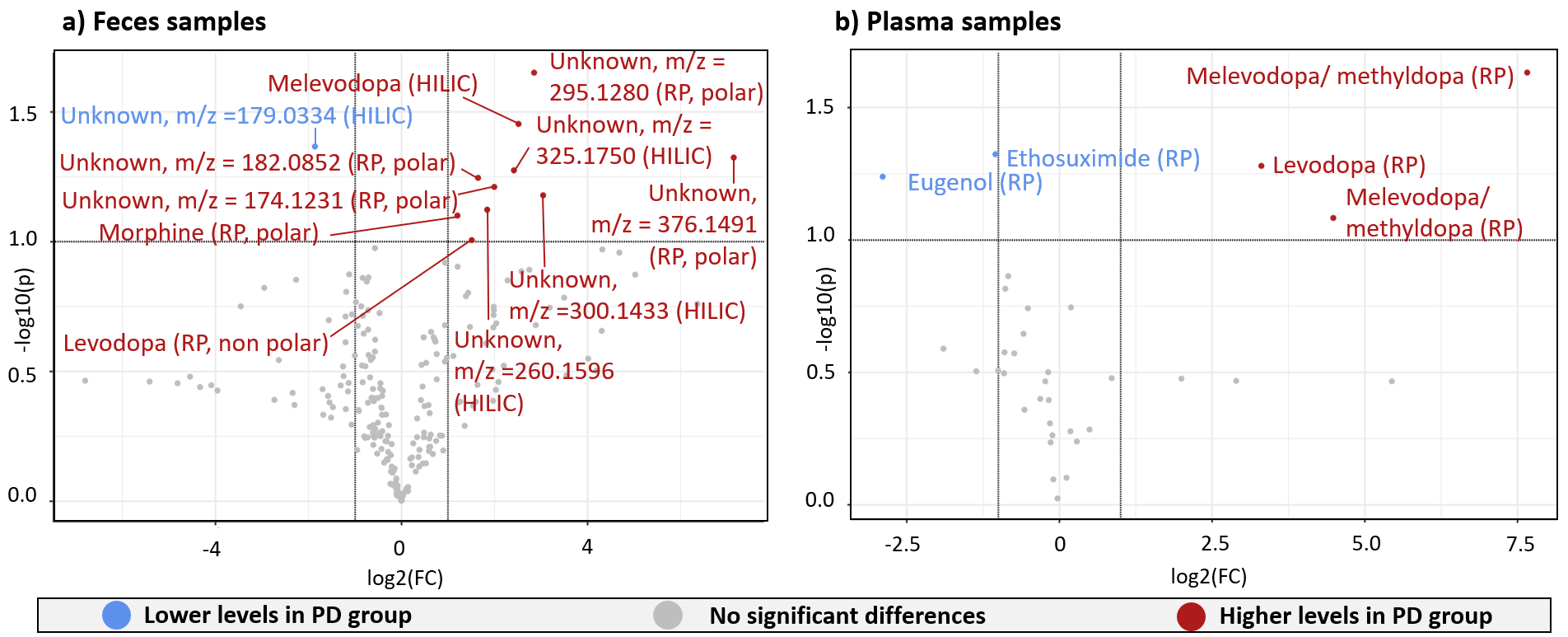


**Fig. S9.** Important features detected in (a) feces and (b) plasma selected by volcano plot with FC threshold (x) 2 and t-tests threshold (y) 0.1. Only features identified by the PD-CTD suspect list are included in the plot. The red circles represent features above the threshold. Note both fold changes and p- values are log transformed. Increasing distance from (0,0) indicates increasing significance.

**References**

1. Benito S, Sánchez-Ortega A, Unceta N, Andrade F, Aldámiz-Echevarria L, Goicolea MA, Barrio RJ (2018) Untargeted metabolomics for plasma biomarker discovery for early chronic kidney disease diagnosis in pediatric patients using LC-QTOF-MS. The Analyst 143:4448–4458. https://doi.org/10.1039/c8an00864g

2. Chen Y, Xu J, Zhang R, Abliz Z (2016) Methods used to increase the comprehensive coverage of urinary and plasma metabolomes by MS. Bioanalysis 8:981–997. https://doi.org/10.4155/bio-2015-0010

3. Bruce SJ, Tavazzi I, Parisod V, Rezzi S, Kochhar S, Guy PA (2009) Investigation of human blood plasma sample preparation for performing metabolomics using ultrahigh performance liquid chromatography/mass spectrometry. Anal Chem 81:3285–3296. https://doi.org/10.1021/ac8024569

4. Southam AD, Haglington LD, Najdekr L, Jankevics A, Weber RJM, Dunn WB (2020) Assessment of human plasma and urine sample preparation for reproducible and high-throughput UHPLC-MS clinical metabolic phenotyping. Analyst 145:6511–6523. https://doi.org/10.1039/d0an01319f

5. Broadhurst D, Goodacre R, Reinke SN, Kuligowski J, Wilson ID, Lewis MR, Dunn WB (2018) Guidelines and considerations for the use of system suitability and quality control samples in mass spectrometry assays applied in untargeted clinical metabolomic studies. Metabolomics 14:72. https://doi.org/10.1007/s11306-018-1367-3

6. Cesbron N, Royer A-L, Guitton Y, Sydor A, Le Bizec B, Dervilly-Pinel G (2017) Optimization of fecal sample preparation for untargeted LC-HRMS based metabolomics. Metabolomics 13:99. https://doi.org/10.1007/s11306-017-1233-8

7. Deda O, Gika HG, Wilson ID, Theodoridis GA (2015) An overview of fecal sample preparation for global metabolic profiling. J Pharm Biomed Anal 113:137–150. https://doi.org/10.1016/j.jpba.2015.02.006

8. Deda O, Gika HG, Theodoridis GA (2018) Rat Fecal Metabolomics-Based Analysis. In: Theodoridis GA, Gika HG, Wilson ID (eds) Metabolic Profiling. Springer New York, New York, NY, pp 149–157

9. Karu N, Deng L, Slae M, Guo AC, Sajed T, Huynh H, Wine E, Wishart DS (2018) A review on human fecal metabolomics: Methods, applications and the human fecal metabolome database. Anal Chim Acta 1030:1–24. https://doi.org/10.1016/j.aca.2018.05.031

10. Moosmang S, Pitscheider M, Sturm S, Seger C, Tilg H, Halabalaki M, Stuppner H (2019) Metabolomic analysis-Addressing NMR and LC-MS related problems in human feces sample preparation. Clin Chim Acta Int J Clin Chem 489:169–176. https://doi.org/10.1016/j.cca.2017.10.029

11. patRoon handbook. https://rickhelmus.github.io/patRoon/handbook_bd/index.html. Accessed 4 Mar 2022
